# Experimental and In Silico Studies to Unravel the Antioxidant and Antibacterial Properties of Lichen Metabolites from *Pseudocyphellaria compar* and *Pseudocyphellaria nudata*

**DOI:** 10.3390/antiox15010034

**Published:** 2025-12-25

**Authors:** Mauricio A. Cuellar, Jessica Mejía, Helena Quintero-Pertuz, Alejandro Castro-Álvarez, Marco Mellado, Waleska Vera-Quezada, Gloria Montenegro, Christian Espinosa-Bustos, Raquel Bridi, Cristian O. Salas

**Affiliations:** 1 Facultad de Farmacia, Escuela de Química y Farmacia, Universidad de Valparaíso, Valparaíso 2340000, Chile; mauricio.cuellar@uv.cl (M.A.C.); waleska.vera@uv.cl (W.V.-Q.); 2 Centro de Investigación, Desarrollo e Innovación de Productos Bioactivos (CINBIO), Universidad de Valparaíso, Valparaíso 2340000, Chile; 3 Departamento de Ciencias Vegetales, Facultad de Agronomía e Ingeniería Forestal, Pontificia Universidad Católica de Chile, Santiago 7820436, Chile; jcmejia@uc.cl (J.M.); gmonten@uc.cl (G.M.); 4 Departamento de Química Farmacológica y Toxicológica, Facultad de Ciencias Químicas y Farmacéuticas, Universidad de Chile, Santiago 8380000, Chile; helena.quintero@ug.uchile.cl; 5 Departamento de Ciencias Preclínicas, Facultad de Medicina, Universidad de La Frontera, Temuco 4780000, Chile; luis.castro@ufrontera.cl; 6 Millennium Nucleus Bioproducts, Genomics and Environmental Microbiology (BioGEM), Valparaíso 2390123, Chile; 7 Centro de Investigación en Ingeniería de Materiales, Universidad Central de Chile, Santiago 8330507, Chile; marco.mellado@ucentral.cl; 8 Departamento de Farmacia, Facultad de Química y de Farmacia, Pontificia Universidad Católica de Chile, Santiago 7820436, Chile; ccespino@uc.cl; 9 Medicinal Chemistry Laboratory, Facultad de Química y de Farmacia, Pontificia Universidad Católica de Chile, Santiago 7820436, Chile

**Keywords:** lichen metabolites, *Pseudocyphellaria*, antioxidants, antibacterials, in silico, RecA protein

## Abstract

Lichens are a source of diverse compounds with a wide range of biological activities, making them of significant interest for novel drug development. In this study, metabolites were extracted from *Lobariaceae* lichens, and their antioxidant and antibacterial properties were experimentally investigated and explained using various computational approaches. Specifically, four lichen metabolites were analyzed using three methods to assess their antioxidant capacity. Antibacterial activity assays were conducted against four pathogens, and the minimum inhibitory concentrations (MICs) of the most promising compounds were determined. Ab initio studies were performed to evaluate radical stability. A pharmacological target responsible for the antibacterial effect was identified, and possible binding sites and modes were studied in silico. Metabolite **IX**, physciosporin, exhibited the highest antioxidant activity, which was associated with the theoretical stability of the radical. Additionally, **IX** exhibited an MIC of 0.97 μg/mL against *S. pyogenes*, surpassing the potency of streptomycin. The RecA protein was identified as a potential target, and a possible binding site and pattern of interactions at that site were described. Finally, **IX** showed low cytotoxicity in human cancer cell lines and was predicted to have favorable oral absorption properties, supporting its potential as a promising antioxidant and antibacterial agent against *S. pyogenes.*

## 1. Introduction

Lichens are symbiotic organisms formed between heterotrophic mycobionts and one or more autotrophic photobionts [1]. Owing to their high ecological plasticity, lichens can colonize most terrestrial habitats [2]. Despite being slow-growing, symbiotic integration produces secondary metabolites known as lichen metabolites [3]. More than 1000 different metabolites have been described [4]. The production of these metabolites involves different biosynthetic pathways, such as acetate polymalonate, shikimic acid, and mevalonic acid [5]. These organisms produce a wide variety of compounds, including dibenzofurans, depsides, depsidones, xanthones, pulvinic acid derivatives, terpenes, and steroids, which exhibit diverse biological activities, such as antimicrobial, antioxidant, and cytotoxic effects [3,6,7,8]. Secondary metabolites of lichens, macrofungi, and vascular plants are significant sources of bioactive substances with antimicrobial activity [9,10,11]. Although lichens constitute 8% of terrestrial ecosystems and there are more than 20,000 species worldwide, their biological activities and bioactive metabolites remain largely unexplored [11,12].

**Figure 1:**
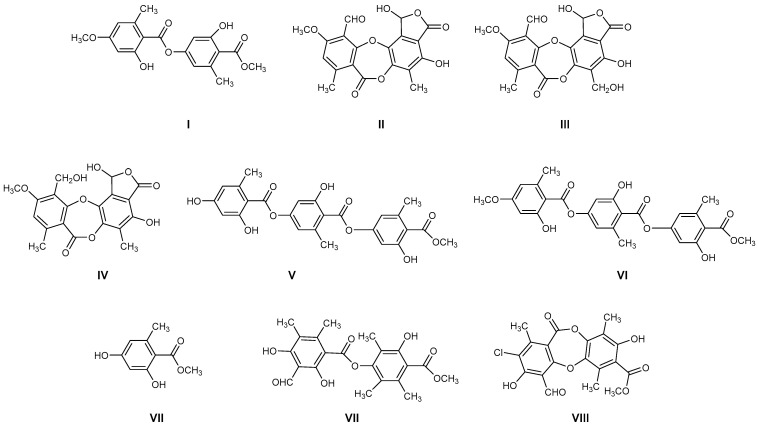
Chemical structures of lichen metabolites extracted from several lichens in Chile.

The most common lichen species belong to the *Lobariaceae* family, with *Pseudocyphellaria* being particularly notable for its diverse secondary chemistry. In Chile, this genus includes 54 documented species. The main lichen metabolites identified in this genus include hopan-6α,7β,22-triol, 2-methoxy-3,6-dimethyl-4-hydroxybenzaldehyde [13], hopan-22-ol, hopan-16β,22-diol, and methyl evernate (**I**, Figure 1) [5]. They have also been identified as stictic acid (**II**), constictic acid (**III**), cryptostic acid (**IV**), and methyl gyrophorates (**V**) [14]. Tenuiorin (**VI**) and methyl orsellinate (**VII**) are common compounds found in *Pseudocyphellaria* and other lichen genera [5,13,15]. In addition, calicin, pulvinic dilactone, pulvinic acid, 22α-hydroxyestictan-3-one, 2α-acetoxyestictan-3β,22α-diol, and pseudocyphellarin A (**VIII**, Figure 1) have been identified [14]. Physciosporin (**IX**, Figure 1) is a lichen metabolite commonly found in the genus *Pseudocyphellaria* [4]. From a chemical perspective, compounds **I**, **V**, **VI**, and **VII** are depsides, while **II–III** and **VII** are depsidones. **VII** is a derivative of benzoic acid, which is a typical monomeric unit of depsides and depsidones.

Among the biological activities of lichens, their ability to inhibit bacterial growth is of great interest [16]. Depsides, depsidones, and tridepsides have recognized antimicrobial properties against Gram-positive bacteria (*Bacillus*, *Staphylococcus*, *Mycobacterium*, *Streptococcus*, and *Enterococcus* genera), Gram-negative bacteria (*Escherichia* and *Proteus* genera), fungi (*Candida* spp.), and parasites (*Plasmodium falciparum* and *Schistosoma mansoni*). It has been reported that the antimicrobial activity varies in potency depending on the type of bacteria, with the highest activity against Gram-positive bacteria [7]. Some of these molecules are RecA inhibitors that enhance bactericidal activity by reducing antibiotic resistance [7,16,17]. The identification of new antibiotics is important because antimicrobial resistance (AMR) is a critical global health concern [18]. This has been driven by the indiscriminate use of antibiotics in both human and animal health, as well as in agriculture, leading to the emergence of resistant microorganisms, which could surpass other causes of mortality by 2050 [19]. Mechanisms such as enzymatic modification and biofilm formation allow microbes to resist the effects of antibiotics [20].

Many phenolic compounds from lichens exhibit antioxidant and antimicrobial properties [21,22]. Antioxidants scavenge and neutralize free radicals, modulating oxidative stress. Given that certain bacteria rely on reactive oxygen species for metabolic or signaling processes, the depletion of these oxidants may contribute to the observed antimicrobial effects [23]. The antimicrobial activity of phenolic compounds is associated with their ability to disrupt bacterial metabolism, primarily by forming complexes with the cell wall. This interaction compromises membrane integrity, leading to structural and functional damage [24,25].

From a biotechnological perspective, lichens are highly valuable species because of their high biodiversity and ability to produce a variety of secondary metabolites. Therefore, when bioactive molecules are discovered, one alternative for their production is chemical synthesis because their cultivation or co-cultivation in the laboratory is difficult due to their slow growth and sensitivity to environmental conditions. In addition, human consumption of some species (*C. islandica*, *C. subgen*, and *Usnea*) poses a major problem, both economically and in terms of nature conservation [26].

**Figure 2:**
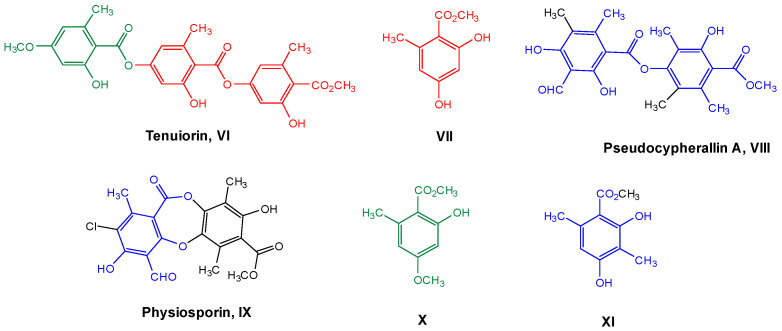
Chemical structures of lichen and monomeric compounds. The colors represent a part of the lichen structure that could be derived from **VII**, **X**, or **XI**.

Therefore, considering all the previous antecedents, we decided to study the antioxidant and antibacterial properties of some metabolites of Chilean lichens, as well as monomers, such as methyl orsellinate **VII** and its analogs **X–XI**, which could be part of the chemical structures of these metabolites (Figure 2, according to the respective colored fragments). The purpose of including the monomers was to understand whether the biological properties elicited by the liquid metabolite could be attributed to these chemical units. Likewise, an in silico exploration of biological targets related to antimicrobial activity was performed to determine the possible mechanism of action. Finally, the cytotoxicity of these metabolites and their predicted oral bioavailability were analyzed to offer a more complete characterization of the most promising compounds as antimicrobial agents based on natural products.

## 2. Materials and Methods

### 2.1. Lichen Material: Extraction and Isolation

*Pseudocyphellaria compar* was collected on the road to Palguín, 5 km from Salto La Mula (−39.371491, −71.783413) (39°22′17.368″ S, 71°47′0.287″ W), Pucón (IX region), Chile, in July 2021. The species was identified by comparing it with samples from the Lichen Herbarium of the Escuela de Química y Farmacia, Universidad de Valparaíso. Voucher for *P. comprar* is COM-0015 and was deposited in the Lichen Herbarium.

The air-dried lichen sample (200 g) was ground and extracted with ethyl acetate (4 L) for 72 h at room temperature. The solvent was removed under reduced pressure to yield an ethyl acetate extract (16.3 g) of the fruit. The extract was chromatographed on silica gel using a mixture of hexane, ethyl acetate/hexane (2% gradient), and methanol to obtain eight fractions. Of the eight fractions, three showed pure compounds, one of which was pseudocyphellarin A (**VIII**, 0.231 g). The remaining five fractions were chromatographed on silica gel or preparative chromatography plates. Three additional compounds were identified: 22α-hydroxisticta-3-one (0.284 g), physciosporin (**IX**, 0.098 g), and calicine (0.300 g).

These compounds were characterized by their melting points and identified using spectroscopic data (^1^H-NMR, ^13^C-NMR, and FT-IR; the respective spectra of these compounds are available in the Supplementary Material Section). These data are consistent with those previously reported [5,13]:

Pseudocyphellarine A (**VIII**) C_21_H_22_O_8_ (m.p. = 173–175 °C)


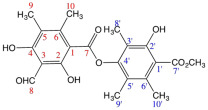


IR (KBr) υ cm^−1^ → 3400–3300 and 1260–1100 (OH), 2850, 2750 and 1725 (CHO), 3020–3000 and 1600, 1475 (Ar), 1750–1735 and 1210–1160 (COOR), 2950–2800 (CH_3_). ^1^H-NMR (CDCl_3_, 400 MHz) → δ: 13.08 (1H, s, OH-2); 12.39 (1H, s, OH-4); 11.12 (1H, s, OH-2′); 10.38 (1H, s, CHO-8); 3.98 (3H, s, 7′-COOMe); 2.70 (3H, s, Me-9); 2.47 (3H, s, Me-9′); 2.19 (3H, s, Me-10); 2.09 (3H, s, Me-8′); 2.07 (3H, s, Me-10′). ^13^C-NMR (CDCl_3_, 50 MHz) → δ: 194.0 (C-8); 172.1 (C-7′); 169.7 (C-7); 166.9 (C-2); 166.1 (C-4); 158.9 (C-2′); 151.5 (C-6); 150.1 (C-4′); 137.6 (C-6′); 120.5 (C-5′); 118.2 (C-5); 116.2 (C-1′); 111.9 (C-3′); 107.9 (C-3); 102.8 (C-1); 52.3 (7′-COOMe); 20.5 (C-10′); 18.8 (C-10); 13.2 (C-9′); 10.8 (C-9); 9.8 (C-8′).

Physciosporin (**IX**) C_19_H_15_ClO_8_ (m.p. = 201–202 °C)


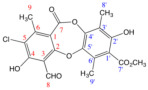


IR (KBr) υ cm^−1^ → 3400–3300 and 1260–1100 (OH), 2850, 2750 and 1725 (CHO), 3020–3000 and 1600, 1475 (Ar), 1750–1735 and 1210–1160 (COOR), 1250 and 1120 (ROR), 2950–2800 (CH_3_). ^1^H-NMR (CDCl_3_, 400 MHz) → δ: 12.84 (1H, s, OH-4); 11.47 (1H, s, OH-2′); 10.71 (1H, s, 8-CHO); 3.96 (3H, s, 7′-COOMe); 2.59 (3H, s, Me-9); 2.57 (3H, s, Me-9′); 2.27 (3H, s, Me-8′). ^13^C-NMR (CDCl_3_, 50 MHz) → δ: 192.8 (8-CHO); 171.3 (C-7′); 162.7 (C-7); 161.3 (C-2); 161.0 (C-4); 159.3 (C-2′); 150.6 (C-4′); 146.8 (C-5′ + C-6); 129.1 (C-6′); 121.2 (C-3′); 117.2 (C-5); 114.2 (C-3); 110.8 (C-1′); 109.3 (C-1); 52.6 (7′-COOMe); 19.8 (C-9); 15.6 (C-9′); 9.3 (C-8′).

### 2.2. Chemicals

Monomers **VII** and **X** were obtained by hydrolyzing tenuiorin (**VI**), which was extracted according to previous work from *Pseudocyphellaria nudata* [5,13]. The hydrolysis of **VI** was carried out using the following procedure. Tenuiorin (1.5 g) was dissolved in 30 mL of a 10% KOH/methanol solution, and the mixture was stirred at room temperature for 24 h. The pH was then adjusted to 7 using HCl. The solvent was evaporated, and the mixture was reconstituted by adding 200 mL ethyl acetate and 200 mL of water. The organic layer was dried using anhydrous Na_2_SO_4_ and concentrated. The residue was purified by silica gel chromatography using a polar mobile phase (AcOEt/hexane 60:40) to obtain **VII** (0.89 g) and **X** (0.54 g). The NMR spectra of the compounds used in this study are provided in the Supplementary Material. Compound **XI** was purchased from Sigma-Aldrich (St. Louis, MO, USA).

### 2.3. Experimental Antioxidant Capacity

#### 2.3.1. Ferric Reducing Antioxidant Potential (FRAP)

The FRAP of the compounds was determined as previously described by our group [27], with minor modifications. The working FRAP solution was prepared daily by mixing 10 parts acetate buffer (0.3 M, pH 3.6), 1 part 10 mM TPTZ (2,4,6-tri(2-pyridyl)-s-triazine, Sigma-Aldrich), and 1 part 20 mM ferric chloride. Aliquots of 270 μL of the FRAP solution were mixed with 30 μL of pure compounds (2.5 mM) diluted 25-fold in MeOH. The reaction mixtures were incubated for 30 min at 37 °C, and absorbance was measured at 594 nm using an AMR-100 plate reader (Hangzhou Allsheng Instruments Co., Ltd., Hangzhou, China). Results were interpolated from a Trolox calibration curve (25–250 µM). Methanol was used as the vehicle control. Antioxidant activity was expressed as µmol Trolox equivalents per gram of compound (µmol TE/g).

#### 2.3.2. ABTS Free Radical Scavenging Activity

ABTS^•+^ activity was measured using a previously reported method [28] with minor modifications. In this study, the ABTS radical (2,2′-azino-*bis*(3-ethylbenzothiazoline-6-sulphonic acid)), which is generated by oxidation with potassium persulfate, was utilized in pure methanolic compound solutions at a concentration of 1.25 mM. A stock solution of ABTS (7.00 mM) was prepared one day before the analysis. The working solution was prepared by diluting the stock solution to an absorbance value of 0.70 ± 0.02 at a wavelength of 734 nm in a quartz cuvette with a volume of 3 mL, using a Helios Gamma spectrophotometer (model UVG 1702E, Thermo Fisher Scientific, Horsham, UK). ABTS working solution (270 µL) was added to each well of the microtiter plate together with 30 µL of the pure compound sample, and the absorbance was determined at 415 nm for 6 min using a Multiskan™ GO UV/Vis microplate spectrophotometer (Thermo Fisher Scientific, Waltham, MA, USA) [29]. The results were expressed as μmol Trolox equivalents per gram of the compound (μmol TE/g).

#### 2.3.3. DPPH Free Radical Scavenging Activity

Antioxidant activity was measured using the method reported in [30], with some modifications, using the DPPH (2,2-diphenyl-1-picrylhydrazyl) radical assay of methanol solutions of pure compounds at a concentration of 1.25 mM. Absorbance was measured at 517 nm using a Multiskan™ GO UV/Vis microplate spectrophotometer (Thermo Fisher Scientific, Waltham, MA, USA) after 60 min in the dark. The results were expressed as μmol Trolox equivalents per gram of the compound (μmol TE/g).

### 2.4. Theoretical Study of Antioxidant Activity

The structures of secondary metabolites (**VI–XI**) were initially drawn using ChemDraw 3D 15.1.0.144 (PerkinElmer Informatics, Inc., Waltham, WA, USA, 1998–2016) and exported in the mol2 format. These files were imported into Gaussian View 5.0.8 (Gaussian Inc., Wallingford, CT, USA, 2000–2008) to configure the theoretical level conditions used to establish the relationship between the antioxidant activity and electronic properties of the compounds.

Geometry optimizations were performed using Density Functional Theory (DFT) calculations with the B3LYP functional and the 6-311+G(d,p) basis set, implemented in Gaussian 09W (Gaussian Inc., Wallingford, CT, USA, 2000–2008), under ideal conditions. For species in the ground state, the restricted model was used, whereas radical species were treated with the unrestricted model, following previously reported methodologies [31]. Additionally, calculations were performed for the reagents used as sensors in the antioxidant assays (ABTS^•+^, DPPH^•^, TPTZ-Fe(III), and their corresponding reduced species). The validity of all optimized structures was confirmed by the absence of any imaginary frequencies. The molecular orbitals were visualized using an isovalue of MO = 0.0200 and density = 0.0004.

To relate the electronic properties to the antioxidant activity, the adiabatic ionization potential (AIP) was calculated as follows:

AIP = E_HOMO_(AOX^•+^) − E_HOMO_(AOX)

Complementarily, a structure-property analysis was performed following previously established guidelines [32]. The electronic properties of the metabolites in their ground state were correlated with the antioxidant activities obtained experimentally for each method (ABTS^•+^, DPPH^•^, and FRAP), using a significance criterion based on Pearson’s correlation coefficient (|r| > 0.7). Additionally, the same analysis was performed considering the radical species of the antioxidants once they had reacted with each reagent to determine their activity. Owing to the mechanistic differences between the antioxidant assays, statistical analyses were performed separately [33].

### 2.5. Antibacterial Activity

The antibacterial activity of the lichen compounds was determined based on the diameter of the growth inhibition zone against *Escherichia coli* (ATCC-25922), *Staphylococcus aureus* (ATCC-25923), *Salmonella typhi* (ATCC-700623), and *Streptococcus pyogenes* (ISP364-00) (Instituto de Salud Pública, Santiago, Chile). The diameter of growth inhibition was determined using the Clinical and Laboratory Standards Institute (CLSI) standard (2006) [34], and bacterial strains were inoculated on Mueller Hinton agar for 24 h at 37 °C. Colonies were then suspended in saline to obtain a turbidity equivalent to 0.5 McFarland (≈1.5 × 10^8^ CFU/mL; Becton Dickinson, Franklin Lakes, NJ, USA). The bacterial suspensions were swabbed uniformly onto Mueller–Hinton agar plates, and wells of 6 mm in diameter were bored into the agar. A 100 μL aliquot of each sample (1 mg/mL in DMSO) was placed in each well. The cultures were incubated for 18–24 h at 37 °C. The diameters of the inhibition zones were measured and compared with those produced by streptomycin [35].

Minimum inhibitory concentrations (MICs) were determined using the standard broth microdilution technique. For the MIC assays, 150 µL of Mueller–Hinton broth and 150 µL of extract (1 mg/mL) were added to the first well, and serial dilutions were performed. Bacterial suspensions were adjusted to approximately 1.5 × 10^8^ CFU/mL (0.5 McFarland standard), and a 50 µL aliquot of each suspension was added to each well. The microplates were then incubated at 37 °C for 24 h. From each well, a 4.5 µL aliquot was taken and seeded onto agar plates. Two negative controls were included, consisting of Mueller–Hinton broth and DMSO, each receiving a 50 µL aliquot of the bacterial suspension. The positive control consisted of streptomycin (1 mg/mL), with a 50 µL aliquot of each bacterial suspension. The lowest concentration that inhibited bacterial growth was recorded as the MIC for each bacterial strain. Antibacterial activity is expressed as inhibition-zone diameter (mm) and MIC (µg/mL). Owing to the semi-quantitative disk diffusion endpoint and the stepwise MIC (two-fold dilution) readout, results were not subjected to inferential statistics.

### 2.6. In Silico Studies: Simulation Ligand-Protein System

#### 2.6.1. Ligand and Protein Preparation

The ligands were created using Avogadro software, version 1.2.0, to obtain the three-dimensional structures of each ligand, assigning the corresponding aromaticity and hydrogen atoms. Subsequently, partial charges were assigned using the QUACPAC program, version 2.2.5.1, and protonations were generated using FixPka, version 2.2.5, at a pH of 7.3. The conformation of each ligand was determined using the Omega software [36].

The RecA monomeric protein was obtained from the AlphaFold Protein Structure Database server (https://alphafold.ebi.ac.uk/, accessed 24 March 2025, ID AF-P0C095-F1-v6) [37]. Energy minimization was performed using 5 ns molecular dynamics simulations to optimize the protein side chains. The quaternary structure of RecA was modeled using AlphaFold 3 [38] from the sequence corresponding to the RecA protein of serotype M1 (UniProt ID P0C095), considering three units of RecA, ADP, Mg^2+^, and the nucleic acid fragment of the *E. coli* RecA crystal (PDB ID 3CMT [39]) as a template to constitute the various monomeric units and assemble the final quaternary structure. This complex was subjected to 5 ns molecular dynamics minimization to improve intermolecular contacts and relax the ATPase domain in the presence of its cofactors and natural ligands. Subsequently, hydrogen atoms were incorporated using the Protein Preparation module of the Schrödinger Suite. In addition, the protonation of basic and acidic amino acid residues was assigned at pH 7.3 using PropKa, version 3.1, and the orientation of the side chains of the residues was adjusted using the OPLS4 force field [39].

#### 2.6.2. Binding Site Detection in RecA Monomer

The SiteMap module of the Schrödinger 2023-3 suite [40] was used to identify potential binding sites in proteins using an energy-based algorithm. The process consists of three sequential steps: (i) cavity detection, (ii) structural characterization, and (iii) evaluation by scoring the results. The detection stage is divided into three phases: first, a grid of points with 1 Å spacing is generated around the protein; then, points that overlap with protein atoms or have low relative confinement (<0.5 by default) are removed. This parameter quantifies the fraction of 110 radial rays emitted from each point on the grid that intercepts the protein surface within 8 Å. The analysis identified five potential binding sites based on the probability of interaction with the small ligands

#### 2.6.3. Molecular Docking

Molecular docking was performed according to the nature of the structural model (monomer or quaternary structure). For the monomeric model, the centroids defined by SiteMap were used as grid centers, and the volume was sized to allow free rotation of the ligands during the conformation search. All docking experiments were performed using Glide in Standard Precision (SP) mode. The best pose for each site was reevaluated using MM-GBSA to refine the affinity estimate by incorporating implicit solvation. In the case of the quaternary structure of RecA (Figure 6B), the grid was centered on the ADP modeled in the ATPase site with a dimension of 20 Å. After docking with Glide SP [41], the poses were grouped into clusters within the extensive cavity. The best poses from each cluster were re-optimized using MM-GBSA [42], and calculations were performed using the Prime program, version 6.2. Given the high affinity energy observed for the ligand at this site, an energy decomposition analysis per residue was performed to identify the key amino acid contributions to protein-ligand interaction.

### 2.7. Cytotoxic Activity

The American Type Culture Collection (ATCC, Rockville, MD, USA) supplied the following experimental cell lines: MCF-7, HT-29, and PC-3. These cell lines were grown in DMEM-F12 containing 10% FCS, 100 U/mL penicillin, 100 μg/mL streptomycin, and 1 mM glutamine. Cells were seeded at 100 μL per well at a plating density of 3 × 10^3^ cells/well and incubated for 24 h at 37 °C in a humidified atmosphere containing 5% CO_2_ to enable attachment. The cells were treated with different concentrations of the synthesized compounds for 72 h under the same conditions. The compound stock solution was prepared in DMSO, and the final concentration of DMSO was maintained at 0.1%. The control cultures were treated with 1% ethanol.

Cells were seeded in 96-well plates at a density of 1 × 10^4^ cells/well. After 24 h, the compounds **VI–XI**, daunorubicin and 5-fluorouracil were added at increasing concentrations (500–100–20–4–0.8–0.16–0.032–0 µM) and the cells were incubated at 37 °C in a humidified 5% CO_2_/95% air mixture for 48 h. Following incubation, the cells were washed twice with PBS, and 100 μL of 0.5 mg/mL MTT solution was added to each well. After 2 h of incubation, the MTT solution was removed, and the formazan crystals were dissolved in 50 μL DMSO per well. Absorbance was measured at 570 nm using a microplate ELISA reader (Thermo Fisher Scientific, Waltham, MA, USA). The IC_50_ values were calculated using SigmaPlot^®^ software (version 11.0) [43].

### 2.8. Statistical Analyses

Statistical analyses were performed using GraphPad Prism 10 (GraphPad Software, San Diego, CA, USA). Data are expressed as mean ± SD from three independent experiments. Normality was assessed using the D’Agostino–Pearson test. Antioxidant activity data were analyzed by one-way ANOVA followed by Tukey’s multiple-comparison post hoc test. Differences were considered statistically significant at *p* < 0.05.

## 3. Results and Discussion

### 3.1. Antioxidant Properties of Lichen Compounds and Monomers

Table 1 summarizes the results obtained from the three complementary assays used to evaluate the antioxidant activity of lichen-derived compounds and monomers. The use of multiple methods is essential, as each assay interrogates different aspects of the antioxidant mechanisms. Specifically, (i) FRAP measures reducing power via single-electron transfer under acidic conditions; (ii) ABTS assesses total antioxidant capacity through a mixed-mode mechanism involving both electron and proton transfer; and (iii) DPPH evaluates radical scavenging capacity based on electron donation to a stable organic radical [44].

As shown in Table 1, physciosporin **IX** demonstrated high antioxidant activity in FRAP and DPPH assays, outperforming **XI** and the other compounds analyzed in DPPH. This superior activity is likely related to its structural characteristics, which enhance its ability to scavenge free radicals and reduce the number of oxidative species. These findings also highlight compound **XI** as the most effective phenolic antioxidant identified in this study, as it reacts more quickly with free radicals than biomolecules (protected molecules) to offer protection against oxidation [45].

### 3.2. Computational Study of Antioxidant Activity

**Figure 3:**
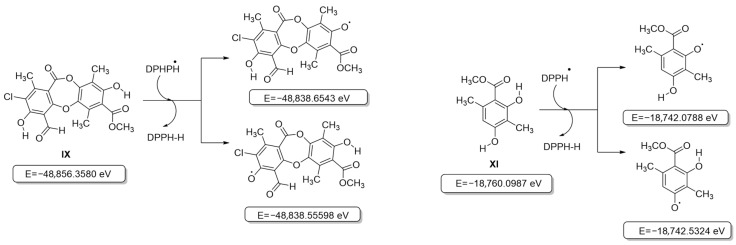
Structures and internal energies of compounds **IX** and **XI** based on the HAT model (DPPH).

Initially, all secondary metabolite structures of the lichen *Pseudocyphellaria compar* were optimized in the gas phase without imaginary frequencies, following the guidelines proposed by Kabanda et al. [31]. Given that antioxidant activity assays are based on different mechanisms—Hydrogen Atom Transfer (HAT, ABTS^•+^ and DPPH^•^ assays) and Single Electron Transfer (SET, FRAP assay) [32], the behavior of the compounds was analyzed independently for each mechanism. The first step between the phenol moiety of the antioxidant and the oxidizing agent (ABTS^•+^ and DPPH^•^ radicals) leads to the formation of a radical species capable of delocalizing the unpaired electron through resonance, which contributes to the stabilization of the generated radical [33]. In this context, the relative energies of the different radicals that can be formed during this process were evaluated by considering compounds **VI–XI** (Tables S1–S5). The results showed that for all the compounds evaluated, the removal of the hydrogen atom from the aromatic –OH group that did not participate in an intramolecular hydrogen bridge was thermodynamically more favorable than the removal from an –OH group involved in such an interaction, in agreement with previous reports [46], and is shown in Figure 3 for the most active compounds **IX** and **XI** (for the other compounds, see Figure S1). Likewise, it was observed that the overall process of transferring the hydrogen atom from the antioxidant to the oxidizing agent to form a stable intermediate is not very favorable, given the increase in the relative energy of all the structures evaluated, including that of the chromophores used in the antioxidant activity tests (Figure S1) [33].

**Figure 4:**
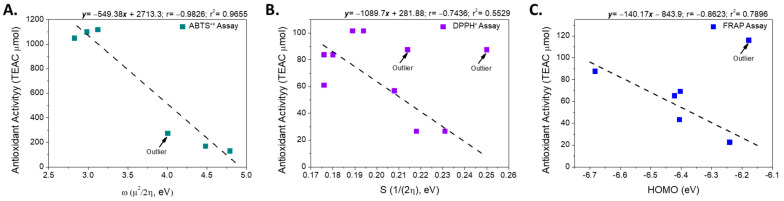
Linear relationship between the electronic descriptors and the antioxidant activity according to assays: (**A**) ABTS^•+^, (**B**) DPPH^•^ and (**C**) FRAP.

Therefore, the electronic properties of the compounds were analyzed using a structure-property relationship model, following an approach similar to that reported previously [32]. First, the electronic descriptors obtained from the calculations in the ground state were used as variables to evaluate the intrinsic reactivity of each free radical analyzed (ABTS^•+^ and DPPH^•^). The analysis revealed that the antioxidant activity exhibited by the metabolites in the ABTS^•+^ assay showed a strong negative correlation with the overall electrophilicity index (ω, r = −0.983, Figure 4), a parameter that describes the stabilization energy associated with the acceptance of a fraction of the electronic charge by a chemical species [47]. This result suggests that in the reaction mechanism involved in the ABTS^•+^ assay, the antioxidant efficiency is mainly determined by the compound’s ability to stabilize the radical formed in the first step of the process [48].

Although both the ABTS^•+^ and DPPH^•^ assays are based on a predominant HAT mechanism, the values obtained using both methods were not correlated (r = 0.244). This difference can be attributed to the energy disparity between the two radicals: the neutralization of ABTS^•+^ requires approximately 2.6 times less energy than that required for DPPH^•^, suggesting different sensitivities to the electronic characteristics of the antioxidant (Figure S1). Considering these results, we evaluated whether the electronic descriptors calculated in the ground state were related to the activity measured using the DPPH^•^ assay. However, no significant correlation was observed (r^2^ < 0.49), indicating that the reactivity in this assay is not governed by the electronic properties of the antioxidant in its neutral state.

Consequently, the relationship between the electronic descriptors of the possible radical species generated after hydrogen atom donation (Figure S1) and the experimental activity was evaluated. This analysis revealed that the DPPH^•^ radical inhibition was inversely correlated with Pearson’s smoothness (r = −0.744, excluding compound **IX**; Figure 3).

This descriptor is associated with the ease of electronic reorganization during the charge transfer process during covalent bonding [49]; that is, those with a lower electronic density distribution or reorganization capacity have higher activity. This suggests that the radicals derived from these compounds tend to retain a higher local electron density, favoring their reactivity with the DPPH^•^ radical.

In the FRAP assay, based on the SET mechanism, the linear relationship between different electronic descriptors and antioxidant activity, expressed in µmol of Trolox equivalents, was evaluated. Considering all the compounds analyzed, no significant correlation was observed between the variables (r^2^ < 0.5). However, when the most active compound (**XI**, FRAP = 116.07 µmol Trolox equivalents) was excluded from the analysis, a strong negative correlation emerged between the energy of the highest occupied molecular orbital (HOMO) and FRAP activity (r = –0.889) (Figure 4).

This correlation indicates that for most of the metabolites analyzed, the antioxidant efficiency observed in the FRAP assay is associated with the ability of the molecule to donate electrons to the Fe(III)–TPTZ complex. From a molecular orbital perspective, efficient electron transfer requires adequate energy compatibility between the donor (HOMO) and acceptor (LUMO) molecular orbitals of the antioxidant and the complex (LUMO).

**Figure 5:**
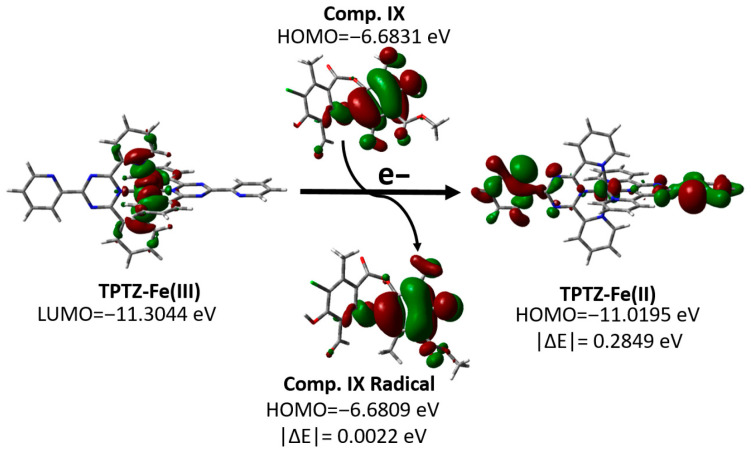
Changes in the molecular orbitals of the FRAP reagent and compound **IX** according to the proposed SET mechanism.

To visualize this process, the HOMO and LUMO orbitals of the most active (**IX**) and least active (**X**) compounds are represented, together with the orbitals involved in the reduction in the Fe(III)–TPTZ complex (Figure S2). As an example of this mechanism, we analyzed the behavior of compound **XI**, one of the most active compounds evaluated, as shown in Figure 5. This figure shows that the LUMO of the Fe(III)–TPTZ complex becomes the HOMO of the reduced Fe (II)–TPTZ complex upon accepting an electron from the antioxidant, with an energy difference in |ΔE| = 0.2849 eV. When these values were compared with the energies of the orbitals of the metabolites evaluated, it was found that the most active compound (**IX**) had a minimal energy difference between its ground-state HOMO and the HOMO of the radical formed (|ΔE| = 0.0022 eV), whereas in the least active compound (**X**), this difference was significantly greater (|ΔE| = 0.2672 eV) (Figure S2). This energy change does not affect the electronic distribution between the ground-state molecular orbitals and the formed radicals. Additionally, this disparity suggests that the efficiency of the SET process in the FRAP assay is strongly conditioned by the energy proximity between the donor orbitals of the metabolites and the acceptor orbitals of the ferric complex, which would explain the low activity observed for compound **X**.

### 3.3. Antibacterial Properties

In this study, the antibacterial activity of six compounds was evaluated against four representative human pathogens: two Gram-positive bacteria (*Staphylococcus aureus* and *Streptococcus pyogenes*) and two Gram-negative bacteria (*Escherichia coli* and *Salmonella typhi*). Table 2 presents the mean diameters of the growth inhibition zones (in mm) for each strain. These values reflect the efficacy of each compound in inhibiting bacterial growth, with larger inhibition zones indicating stronger antibacterial activity.

As shown in Table 2, compounds **VII**, **VIII**, **IX**, and **XI** were selected based on the diameters of the growth inhibition zones in the agar disk diffusion test, and the MIC was determined using a standard microdilution technique. The three lichen metabolites elicited antibacterial properties, and the non-lichen monomer, **XI**.

As shown by the MIC values in Table 3, the results obtained for *S. pyogenes* are particularly noteworthy. This bacterium is a common human pathogen implicated in a spectrum of diseases ranging from mild infections, such as pharyngitis and impetigo, to life-threatening conditions, including necrotizing fasciitis, sepsis, and toxic shock syndrome [50]. The increasing resistance of *S. pyogenes* to multiple antibiotic classes, particularly macrolides and lincosamides, underscores the need to identify alternative therapeutic agents. Resistance to erythromycin, clindamycin, and tetracycline have been reported in several strains, raising clinical concerns. Although penicillin remains the first-line treatment, the emergence of resistance to other antibiotics highlights the need for continued surveillance and novel antimicrobial strategies targeting this pathogen [51].

### 3.4. In Silico Studies for Target Prediction and Binding Modes of **IX**

To explore the possible targets involved in the antibacterial activity of physciosporin **IX**, an in silico study was performed using the Plato r35 web server (https://prometheus.farmacia.uniba.it/plato, accessed 14 March 2025). Plato r35 is an easy-to-use polypharmacology prediction platform designed to identify new putative protein targets for drugs and quantify the affinity value of bioactivity [52,53,54]. The results of this target search yielded several human proteins, as well as a bacterial protein, RecA. Therefore, this was selected as a potential target for the antibacterial activity of physciosporin against *S. pyogenes*. According to some studies, lichen compounds are RecA inhibitors that can enhance antibacterial activity by reducing antibiotic resistance.

#### 3.4.1. In Silico Studies on RecA

**Figure 6:**
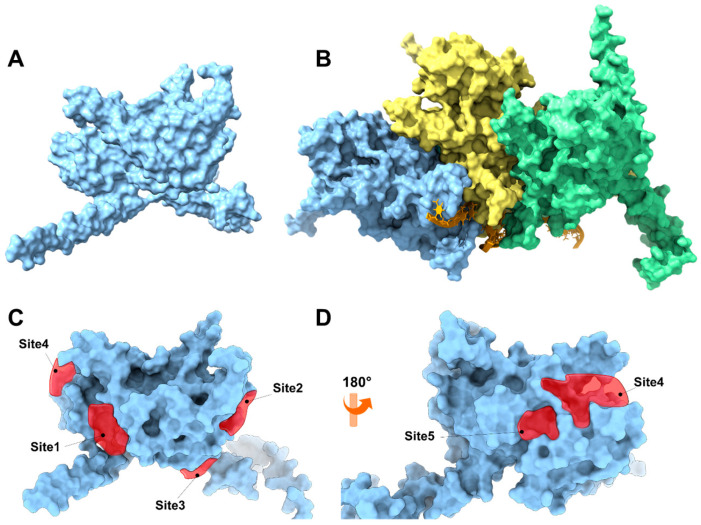
(**A**) Three-dimensional representation of the monomeric RecA unit in *S. pyogenes*. (**B**) Representation of the RecA system with DNA fragments based on the 3CMT template of *E. coli*. The system consisted of three RecA units (light blue, yellow, and green), an orange DNA strand, and ADP in the ATP-binding domain of the RecA protein. The binding sites are represented by red polyhedra. (**C**) Front view of the RecA protein, which is colored light blue, and (**D**) back view of the RecA protein.

An in silico study of the RecA protein from *S. pyogenes* was performed using two strategies: The first strategy focused on analyzing the monomeric form of the protein (Figure 6A) under the hypothesis that physciosporin **IX** exerts its action by inhibiting or sequestering its polymerization similarly to how the RecA protein does [55] or other natural products that inhibit the polymerization of other polyproteins, such as colchicine with tubulin or phalloidin with actin G. In the second approach, **IX** was directed to inhibit the catalytic activity of the ATPase domain of the RecA system (a trimer as a model, Figure 6B), which plays an essential role in activating the bacterial SOS system. For the first strategy (Figure 6A), the monomeric three-dimensional structure of RecA was downloaded from the AlphaFold Protein Structure Database server (https://alphafold.ebi.ac.uk, accessed 24 March 2025, ID AF-P0C095-F1-v6) (Figure S3). Subsequently, the three-dimensional structure of the protein underwent a relaxation process using molecular dynamics simulation for 5 ns to relax the side chains and adjust the dihedral angles of the protein’s backbone. The SiteMap program, version 2.1 [40] was used to analyze the surface of the protein, which allowed the identification of five possible binding sites (Figure 6C,D). Most of these sites correspond to the surface-binding sites. The results of the identified binding sites are listed in Table S6. The SiteMap program does not correctly identify the ATPase domain of the protein because this domain is a pocket formed between the subunits of the monomeric structure, which constitutes its quaternary structure. To better simulate the ATPase domain, the quaternary structure (Figure 6B) was modeled using AlphaFold 3 [37] from the sequence corresponding to the RecA protein of serotype M1 (UniProt ID P0C095).

#### 3.4.2. Molecular Docking with the RecA Monomer

**Figure 7:**
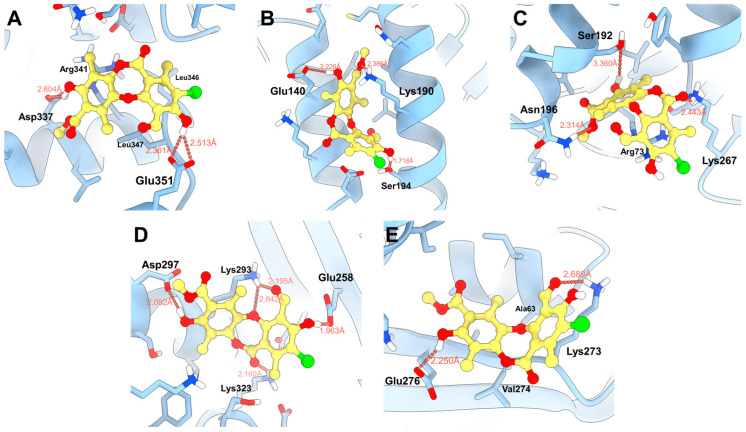
Proposed binding modes of **IX** in the respective cavities: (**A**) site 1, (**B**) site 2, (**C**) site 3, (**D**) site 4, and (**E**) site 5.

Molecular docking studies were performed for the five binding sites identified with physciosporin **IX**, considering the first ten poses for each site (Figure S4). Both the maximum affinity value and the average of each pose, as well as the average affinity energy of all poses, were analyzed. Initially, **IX** showed a higher affinity for binding site 4, with an affinity energy of −4.438 kcal/mol. As shown in Figure 7D, the hydroxyl groups of **IX** formed bonds with Asp297 at 2.082 Å and Glu258 at 1.963 Å. There are additional interactions with Lys293 that establish bonds with the aldehyde carbonyl and cyclic ether of the central core of **IX**. In addition, the carbonyl group of the lactone region interacted with Lys323 at 2.160 Å, contributing to the high affinity observed in the docking studies.

The second binding site with the highest affinity in molecular docking was site 2, located between two alpha helices (Figure 7B), where the natural product formed hydrogen bonds at 2.386 Å with Lys190, 2.226 Å with Glu140, and 1.718 Å with Ser194, respectively. The predominant interactions were established with the hydroxyl and ester groups of the compound, taking advantage of the aromatic and flat regions of the ligand at the binding sites. In contrast, at site 3 (−4335 kcal/mol, Figure 7C), two hydrogen bonds with Lys267 and Asn196 were observed, in addition to a weak interaction with Ser192 at 3360 Å. Furthermore, π–stacking interactions with Arg73 were observed.

MM-GBSA calculations were performed to corroborate the values obtained from the molecular docking studies. This type of analysis improved the estimation of affinity energy by considering the solvation of the ligand and receptor independently using the generalized Born surface area (GBSA) model. In addition, the interactions of amino acid side chains located approximately 6 Å from the center of mass of the bound ligand were included.

The highest affinity energy was obtained for the ligand at site 2, with a value of –40.030 kcal/mol, reflecting greater interaction with residues located in the alpha helices at that site. The second highest affinity was observed at site 5, with an energy of −33.190 kcal/mol, where interactions alternated between Lys273 and the aldehyde and hydroxyl groups of the natural product. Finally, site 1 had the third-best affinity, with an energy of −29,310 kcal/mol, mainly reinforced by the interaction with the Glu351 residue. The obtained energies are listed in Table 4.

#### 3.4.3. Molecular Docking with RecA Complex

**Figure 8:**
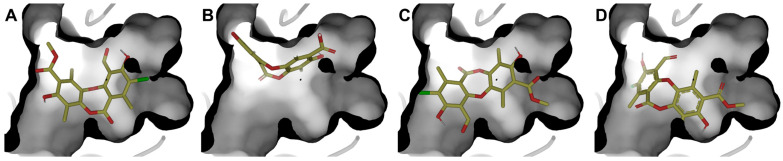
The four best poses were obtained at the ATPase domain binding site. (**A**) pose 1, (**B**) pose 2, (**C**) pose 3, and (**D**) pose 4, with molecular docking calculations.

Authors such as Zhou [56], Alam [17], Kiran [57], among others, point out that the inhibition of RecA activity is mainly due to the inhibition of the ATPase active site, composed of residues Glu81, Lys85, Glu109, Tyr116, Asp157, and Gln207. Therefore, a RecA complex was constructed in AlphaFold 3 considering three units of RecA, ADP, Mg^2+^, and nucleic acid (Figures S5 and S6). The system was refined with 5 ns minimization to improve the side-chain arrangement. Molecular docking was performed at the ADP-binding site. Four poses (Figure 8) of physciosporin at the binding site were identified.

First, it is understandable that the affinity of ADP is lower than that of **IX** because the protein needs to exchange ADP for ATP, a substrate that has a higher affinity for the ATP-binding domain of the protein. However, ADP is conserved because of the crystallographic information contained in the 3CMT template. Poses 1 and 3 showed the highest affinity for the binding site (Table 5), with the most decisive interactions being those due to residues Gly84, Thr86, Asp113, and Ala269 for cluster 1, while cluster 3 showed almost the same residues with the difference that Gly84 was replaced by Thr87 and Ala111 in cluster 3 (Figure S6).

**Figure 9:**
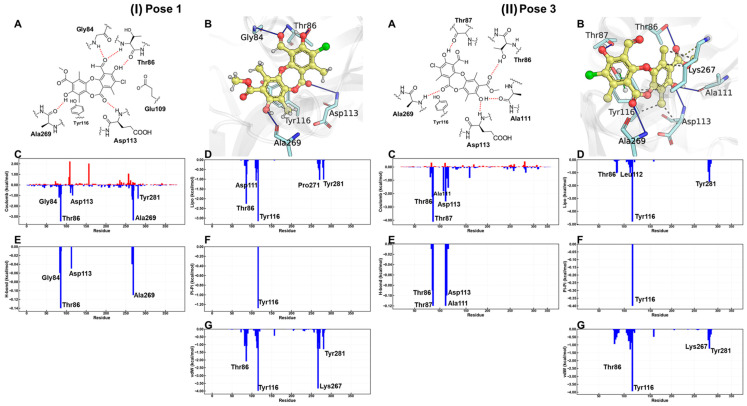
For (**I**) pose 1 and (**II**) pose 3, bidimensional and tridimensional representations of physciosporin **IX** in the ATP site (**A**,**B**). Plots (**C**–**G**) show the MM-GBSA energy decomposition analysis.

According to the MM-GBSA energy decomposition analysis (Figure 9), the affinity of physciosporin **IX** for the binding site in both clusters is predominantly attributed to Coulombic interactions, which originate from a network of hydrogen bonds that anchor the ligand to the ATP-binding pocket. In Cluster 1, hydrogen bonds formed with residues Thr86 and Ala269 were prominent. In Cluster 3, this interaction was mediated by Thr87 and Thr86. In addition, a significant contribution is observed from residue Tyr116, which establishes a π-stacking with one of the aromatic rings of **IX**, regardless of the posture adopted. In contrast, hydrophobic interactions, quantified through van der Waals (vdW) and lipophilic (Lipo) energies, were comparable for both orientations, suggesting a similar contribution of these forces to the stability of the complex. In addition, a significant contribution was observed from the residue Tyr116, established a π–π stacking interaction king with one of the aromatic rings of **IX**, adopted posture.

Despite the similarities, the detailed energy analysis in Figure 9, particularly regarding Coulomb energy, shows that interactions with Thr86 and Ala269 are energetically more favorable in pose 1 conformation than in pose 3. However, it is essential to note that the posture adopted by pose 1 simultaneously presents an unfavorable interaction that imposes an energy penalty on the overall positioning within the ATP-binding site.

These results suggest that physciosporin inhibits the essential catalytic activity of the bacterial protein RecA. This inhibition interferes with intrinsic processes of the protein, such as its binding DNA or its ability to repair damage to genetic material.

### 3.5. Cytotoxic Effect on Cells

Considering the antioxidant and antibacterial properties of physciosporin **IX**, the next step was to determine the effect of this metabolite on mammalian cancer cell lines to estimate its potential cytotoxicity. To do this, viability assays were performed on the three cancer cell lines to evaluate the cytotoxic properties of **IX**. Daunorubicin and 5-fluorouracil (5-FU) were used as the reference drugs. According to the IC_50_ values shown in Table 6, **IX** elicited lower toxicity than daunorubicin or 5-FU in the three cells lines, which could indicate low toxicity in mammalian cells.

### 3.6. Calculated Physicochemical and ADME Properties

**Figure 10:**
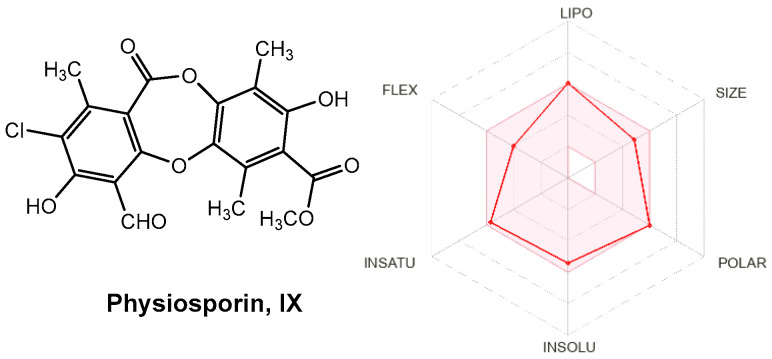
Radar plots of the bioavailability of **IX**. The pink area indicates the range of optimal values for each property related to oral bioavailability. The predicted properties are represented by red lines.

Drug-like properties, such as pharmacokinetic (ADME) and pharmacodynamic (e.g., toxicological) profiles, are important during drug discovery and development. These properties demonstrate the optimization of a leading compound as a successful candidate in the preclinical stages [58]. ADMET properties are important for determining chemical descriptors, such as the polar surface area (PSA) and molecular weight (MW) of molecules, which are useful for determining the oral absorption of drugs. Small hydrophilic molecules undergo rapid renal clearance, whereas large hydrophobic compounds undergo extensive hepatic metabolism and poor absorption [59]. Therefore, finding a suitable hydrophilic-hydrophobic drug balance is a significant challenge for medicinal chemists. Thus, to evaluate these properties and predict good oral bioavailability, two sets of rules, Lipinski and Veber, should be followed for a good prediction [60]. Lipinski’s rule of five states that an orally bioavailable molecule should not violate the following criteria: ≤5 hydrogen bond donors (HBD), ≤10 hydrogen bond acceptors (HBA), MW < 500, and log *p*-value < 5. In contrast, Veber et al. described the role of PSA and the number of rotatable bonds as criteria for estimating oral bioavailability. Veber’s rule states that a compound that is orally bioavailable should have either a PSA ≤ 140 Å and ≤10 rotatable bonds (NRB) or ≤12 HBD and HBA in total and ≤10 rotatable bonds. As shown in Table 7, physciosporin **IX** achieved the drug-likeness criteria described by Lipinski and Veber; therefore, they are expected to have good oral bioavailability. In addition, from SwissADME (http://www.swissadme.ch/index.php, accessed on 17 May 2025), the bioavailability radar chart showed that **IX** is in the desired range (pink region) of five parameters from the six parameters used for oral absorption prediction: FLEX (flexibility), LIPO (lipophilicity), INSOLU (solubility), SIZE, and POLAR (polarity), while they are in the undesirable area of INSATU (saturation) (Figure 10), confirming their good oral bioavailability.

## 4. Conclusions

Four lichen metabolites were isolated from lichen species belonging to the *Lobariaceae* family, and two hydrolysis products were obtained to investigate the structural features underlying their antioxidant activity using ABTS^•+^, DPPH^•^ and FRAP assays. The results indicated that metabolite **IX**, physciosporin, and its monomer **XI** showed the highest antioxidant activity, which can be attributed to the electronic properties of the compounds in both their ground and radical states. Overall, these findings suggest that **IX** and **XI** may act at early stages of the oxidative process. In the ABTS^•+^ and DPPH^•^ assays (HAT-based mechanisms), the antioxidant effect is likely associated with stabilization of the unpaired electron generated upon interaction with the radical species. In contrast, in the FRAP assay (SET-based mechanism), the antioxidants may promote electron transfer from the highest occupied molecular orbital (HOMO) to the oxidizing agent. Furthermore, physciosporin exhibited a notable and selective antibacterial effect against *S. pyogenes* (MIC = 0.7 µg/mL), a pathogen of significant concern because of its resistance to conventional antibiotics and was more potent than streptomycin in this assay. To elucidate the molecular basis of this activity, a potential target was examined, with the RecA protein emerging as a promising candidate. Computational studies indicated that physciosporin is a promising inhibitor of the bacterial protein RecA. MM-GBSA analysis not only identified site 2 as a high-affinity binding site in the monomeric form of RecA but also revealed that physciosporin binds to the ATPase active site with a more favorable binding free energy (−79.77 kcal/mol) than the natural substrate ADP (−66.34 kcal/mol). This strong binding is stabilized by key interactions, including a network of hydrogen bonds and π–π stacking with Tyr116. By competitively occupying this essential catalytic site, **IX** is predicted to impair RecA function, thereby potentially inhibiting vital processes in bacteria, such as DNA repair. Moreover, **IX** showed low cytotoxicity in the three cancer cell lines compared with daunorubicin and 5-fluorouracil, and in silico ADME predictions indicated that **IX** has a favorable pharmacokinetic profile for oral administration.

Therefore, this study supports the hypothesis that lichens are a rich source of bioactive compounds for drug discovery programs. To this end, knowing the chemical structures of these compounds will enable their production by chemical synthesis, due to imitating lichen symbiosis in the laboratory requires large amounts of biomass to produce lichen metabolites.

## Figures and Tables

**Table 1:** FRAP, ABTS, and DPPH values of the compounds in this study.

Compound	FRAP	ABTS	DPPH
**VI**	65.05 ± 0.03 ^a^	273.42 ± 3.60 ^a^	26.60 ± 1.80 ^a^
**VII**	69.14 ± 1.44 ^b^	1118.39 ± 7.74 ^b^	83.88 ± 1.49 ^b^
**VIII**	43.35 ± 0.58 ^c^	169.01 ± 22.77 ^c^	56.92 ± 2.33 ^c^
**IX**	87.57 ± 1.93 ^d^	130.84 ± 2.82 ^d^	110.49 ± 0.35 ^d^
**X**	22.59 ± 0.20 ^e^	1049.68 ± 3.64 ^e^	61.00 ± 1.45 ^c^
**XI**	116.07 ± 0.41 ^f^	1099.51 ± 4.83 ^b^	101.57 ± 10.08 ^d^

FRAP, ABTS, and DPPH as μmol Trolox equivalents (TE)/g. Compounds **VI–XI** were evaluated at 2.5 mM. The values are the mean ± standard deviation from three independent assays. A one-way ANOVA was performed separately for each parameter, followed by Tukey’s multiple comparisons test (*p* < 0.05). Different letters indicate statistical differences within each column.

**Table 2:** Antimicrobial activity against *E. coli*, *S. aureus*, *S. typhi*, *S. pyogenes* expressed as the diameter (mm) of the growth inhibition zone.

Compound	*S. pyogenes* ^a^	*E. coli* ^a^	*S. typhi* ^a^	*S. aureus* ^a^
**VI**	*-*	*-*	*-*	*-*
**VII**	13 mm	12 mm	13 mm	-
**VIII**	13 mm	-	-	-
**IX**	22 mm	-	-	8 mm
**X**	-	-	-	-
**XI**	14 mm	10 mm	11 mm	-

^a^ Antimicrobial activity was expressed as the diameter of the growth-inhibition zone (mm). Values are the mean of three determinations; SD was < 5%. “-“ indicates no inhibition zone was observed.

**Table 3:** Antimicrobial activity against *E. coli*, *S. aureus*, *S. typhi*, *S. pyogenes* expressed as MIC for selected compounds.

	MIC (μg/mL)			
Compound	*S. pyogenes*	*E. coli*	*S. typhi*	*S. aureus*
**VII**	500	500	500	500
**VIII**	7.81	500	500	500
**IX**	0.97	500	500	500
**XI**	250	500	500	500
**Streptomycin**	15.62	31.25	-	31.25

MIC (µg/mL) determined by broth microdilution (two-fold dilutions). “-“ indicates no inhibition was observed under the assay conditions.

**Table 4:** Affinity energies were achieved using molecular docking (Score max, SP average, and SD) and MM-GBSA. All values are expressed in kcal/mol.

Site	Score_max_	SP_average_	SD	MM-GBSA
**1**	−3.864	−3.639	0.116	−29.310
**2**	−4.360	−4.221	0.112	−40.030
**3**	−4.335	−3.477	0.766	−21.110
**4**	−4.438	−4.208	0.225	−20.120
**5**	−3.650	−3.314	0.375	−33.190

Score_max_: Maximum score achieved with a glide (SP Score). SP_average_: Average affinity energy of 10 poses in the binding site of the target protein. SD: Standard deviation.

**Table 5:** Affinity energies achieved by molecular docking (number of clusters, number of ligands poses per cluster, Score max, SP average, and SD) and MM-GBSA. All values are expressed in kcal/mol.

Compound	Pose	Score SP	MM-GBSA
**IX**	1	−6.397	−74.940
	2	−6.204	−60.940
	3	−6.016	−79.770
	4	−5.202	−68.730
**ADP**		−10.213	−66.340

**Table 6:** Cytotoxic activities of **IX** in three cancer cell lines.

Compound	IC_50_ (μM) ^a^		
	MCF-7	HT-29	PC-3
**IX**	31.6 ± 6.9	32.7 ± 7.2	60.7 ± 4.9
**Daunorubicin**	0.33 ± 0.02	15.11 ± 0.5	0.41 ± 0.04
**5-FU**	22.3 ± 0.2	2.9 ± 0.7	16.4 ± 0.6

^a^ IC_50_ values were determined in three separate experiments, each conducted in triplicate. The results are expressed as the IC_50_ ± SD. One-way ANOVA for compound **IX** vs. streptomycin parameter, followed by Dunnett’s multiple comparisons test (*p* < 0.05) concluding that these values are significantly different.

**Table 7:** Molecular properties of physciosporin **IX**.

Compound	MW (Da)	HBA	HBD	cLogP	TPSA (Å^2^)	NRB
Desirable value	≤500	≤10	≤5	≤5	≤140	≤10
**IX**	406.77	8	2	3.39	119.36	3

MW, molecular weight; HBA: Number of hydrogen bond acceptors; HBD: Number of hydrogen bond donors; cLogP, consensus Log *p* value; TPSA: Topological polar surface area; NRB, number of rotatable bonds. All these parameters were predicted by SwissADME (http://www.swissadme.ch/index.php, accessed on 17 May 2025).

## Data Availability

The original contributions presented in this study are included in the article and Supplementary Material. Further inquiries can be directed to the corresponding authors.

## Supplementary Materials

The following supporting information can be downloaded at https://www.mdpi.com/article/10.3390/antiox15010034/s1, IR, 1H, and 13C NMR spectra of selected compounds. Table S1: Energy differences between the ground state and radical state of compound VI. Table S2: Energy differences between the ground state and radical state of compound VII. Table S3: Energy differences between the ground state and radical state of compound VIII. Table S4: Energy differences between the ground state and radical state of compound X. Table S5: Energy differences between the ground state and radical state of chromophores used in DPPH and ABTS assays. Figure S1: Structures of the metabolites analyzed and the energy of each molecule in its ground state and radical state studied as an antioxidant agent based on the HAT model, together with the chromophores used for the tests, Figure S2: Molecular orbitals (HOMO and LUMO), energies and energy differences of the most active (IX) and least active (X) compounds together Fe–TPTZ complex based in a SET mechanism. Table S6: Values obtained with SiteMap. Figure S3: Potential binding sites. (A) Secondary structure of the RecA monomeric unit, (B) the surface of the protein, Figure S4: RecA protein monomer (blue) and physciosporin (represented by yellow spheres) at the different binding sites obtained with SiteMap. Figure S5: Graphical representation of the trimer model with a transparent surface to highlight (circled) the regions corresponding to the ATPase domain. Figure S6: Representation of all poses obtained with molecular docking.

## References

[1] Dembitsky V.M., Shukla V., Kumar S., Kumar N. (2017). The Multiple Properties of Some of the Lichenized Ascomycetes: Biological Activity and Active Metabolites. Plant Adaptation Strategies in Changing Environment.

[2] Kappen L. (2000). Some aspects of the great success of lichens in Antarctica. Antarct. Sci..

[3] Boustie J., Tomasi S., Grube M. (2011). Bioactive lichen metabolites: Alpine habitats as an untapped source. Phytochem. Rev..

[4] Huneck S., Yoshimura I., Huneck S., Yoshimura I. (1996). Identification of Lichen Substances. Identification of Lichen Substances.

[5] Fritis M.C., Rubio L.C., Quiñones S.N., Montenegro V.I., Salas S.C., Carrasco A.H., Espinoza C.L., Palma W.Q. (2013). Depsides and triterpenes in *Pseudocyphellaria coriifolia* (lichens) and biological activity against *Trypanosoma cruzi*. Nat. Prod. Res..

[6] Shukla V., Joshi G.P., Rawat M.S.M. (2010). Lichens as a potential natural source of bioactive compounds: A review. Phytochem. Rev..

[7] Isabel U.-V., Elena González B., Pradeep Kumar D., Maria Pilar G.-S. (2021). Dibenzofurans from Lichens—A Pharmacological Overview. Curr. Top. Med. Chem..

[8] Ramawat K., Merillon J.M. (2007). Biotechnology: Secondary Metabolites Plants and Microbes.

[9] Hostettmann K., Wolfender J.-L. (1997). The search for biologically active secondary metabolites. Pestic. Sci..

[10] Ingolfsdottir K., Hjalmarsdottir M.A., Sigurdsson A., Gudjonsdottir G.A., Brynjolfsdottir A., Steingrimsson O. (1997). In vitro susceptibility of *Helicobacter pylori* to protolichesterinic acid from the lichen *Cetraria islandica*. Antimicrob. Agents Chemother..

[11] Srivastava P., Upreti D.K., Dhole T.N., Srivastava A.K., Nayak M.T. (2013). Antimicrobial Property of Extracts of Indian Lichen against Human Pathogenic Bacteria. Interdiscip. Perspect. Infect. Dis..

[12] Toma N., Ghetea L., Nitu R., Corol D. (2001). Progress and perspectives in the biotechnology of lichens. Roum. Biotechnol. Lett..

[13] Cuellar M., Quilhot W., Rubio C., Soto C., Espinoza L., Carrasco H. (2008). Phenolics, depsides and triterpenes from the chilean lichen *Pseudocyphellaria nudata* (Zahlbr.) DJ Galloway. J. Chil. Chem. Soc..

[14] Galloway D.J. (1992). Studies in *Pseudocyphellaria* (lichens) III. The South American species. Bibl. Lichenol..

[15] Culberson W.L. (1977). Recent Literature on Lichens. 97. Bryologist.

[16] Bellio P., Di Pietro L., Mancini A., Piovano M., Nicoletti M., Brisdelli F., Tondi D., Cendron L., Franceschini N., Amicosante G. (2017). SOS response in bacteria: Inhibitory activity of lichen secondary metabolites against *Escherichia coli* RecA protein. Phytomedicine.

[17] Alam M.K., Alhhazmi A., DeCoteau J.F., Luo Y., Geyer C.R. (2016). RecA Inhibitors Potentiate Antibiotic Activity and Block Evolution of Antibiotic Resistance. Cell Chem. Biol..

[18] Ben Y., Fu C., Hu M., Liu L., Wong M.H., Zheng C. (2019). Human health risk assessment of antibiotic resistance associated with antibiotic residues in the environment: A review. Environ. Res..

[19] Madden J., Outterson K. (2023). Trends in the global antibiotics market. Nat. Rev. Drug Discov..

[20] Hajhamed N.M., Mohamed N.S., Abdalla A.E., Ebrahim R.M.A., Mohammed S.I., Bakheit A.M., Azhary A., Ahmed A.E., Abdelbagi A., Ali M.S.E. (2025). Current status and recent trends in innovative tactics and the One Health approach to address the challenge of methicillin-resistant *Staphylococcus aureus* infections: A comprehensive review. Discov. Med..

[21] Urena-Vacas I., Gonzalez-Burgos E., Divakar P.K., Gomez-Serranillos M.P. (2023). Lichen Depsides and Tridepsides: Progress in Pharmacological Approaches. J. Fungi.

[22] Urena-Vacas I., Gonzalez-Burgos E., Divakar P.K., Gomez-Serranillos M.P. (2022). Lichen Depsidones with Biological Interest. Planta Med..

[23] Silva V., Silva A., Ribeiro J., Aires A., Carvalho R., Amaral J.S., Barros L., Igrejas G., Poeta P. (2023). Screening of Chemical Composition, Antimicrobial and Antioxidant Activities in Pomegranate, Quince, and Persimmon Leaf, Peel, and Seed: Valorization of Autumn Fruits By-Products for a One Health Perspective. Antibiotics.

[24] Mace S., Truelstrup Hansen L., Rupasinghe H.P.V. (2017). Anti-Bacterial Activity of Phenolic Compounds against Streptococcus pyogenes. Medicines.

[25] Kohanski M.A., Dwyer D.J., Hayete B., Lawrence C.A., Collins J.J. (2007). A Common Mechanism of Cellular Death Induced by Bactericidal Antibiotics. Cell.

[26] Zakeri Z., Junne S., Jager F., Dostert M., Otte V., Neubauer P. (2022). Lichen cell factories: Methods for the isolation of photobiont and mycobiont partners for defined pure and co-cultivation. Microb. Cell Fact..

[27] Bridi R., Atala E., Pizarro P.N., Montenegro G. (2019). Honeybee Pollen Load: Phenolic Composition and Antimicrobial Activity and Antioxidant Capacity. J. Nat. Prod..

[28] Concepcion-Alvarez A., Arias-Sante M.F., Hidalgo M., Railef B., Rincon-Cervera M.A., Bridi R., de Alencar S.M., Porras O., de Camargo A.C. (2025). Insoluble-bound phenolics from calafate byproducts: Impact on redox status and oxidative protection in Caco-2 cells. Food Res. Int..

[29] Prior R.L., Wu X., Schaich K. (2005). Standardized Methods for the Determination of Antioxidant Capacity and Phenolics in Foods and Dietary Supplements. J. Agric. Food Chem..

[30] De Camargo A.C., Vieira T.M.F.d.S., Regitano-D’Arce M.A.B., Calori-Domingues M.A., Canniatti-Brazaca S.G. (2012). Gamma Radiation Effects on Peanut Skin Antioxidants. Int. J. Mol. Sci..

[31] Kabanda M.M. (2015). A theoretical study of the antioxidant properties of phenolic acid amides investigated through the radical-scavenging and metal chelation mechanisms. Eur. Food Res. Technol..

[32] Mellado M., Madrid A., Martínez Ú., Mella J., Salas C.O., Cuellar M. (2018). Hansch’s analysis application to chalcone synthesis by Claisen–Schmidt reaction based in DFT methodology. Chem. Pap..

[33] Christodoulou M.C., Orellana Palacios J.C., Hesami G., Jafarzadeh S., Lorenzo J.M., Domínguez R., Moreno A., Hadidi M. (2022). Spectrophotometric Methods for Measurement of Antioxidant Activity in Food and Pharmaceuticals. Antioxidants.

[34] Clinical and Laboratory Standards Institute (2006). Performance Standard for Antimicrobial Disk Susceptibility Testing: Approved Standard.

[35] Jorquera B., Mayorga A., Quintero-Pertuz H., Mejía J., Núñez G., Núñez Pizarro P., Arias-Santé M.F., Montenegro G., Costa de Camargo A., Bridi R. (2023). Phenolics from Chilean Bee Bread Exhibit Antioxidant and Antibacterial Properties: The First Prospective Study. Chem. Biodivers..

[36] Hawkins P.C., Skillman A.G., Warren G.L., Ellingson B.A., Stahl M.T. (2010). Conformer generation with OMEGA: Algorithm and validation using high quality structures from the Protein Databank and Cambridge Structural Database. J. Chem. Inf. Model..

[37] Abramson J., Adler J., Dunger J., Evans R., Green T., Pritzel A., Ronneberger O., Willmore L., Ballard A.J., Bambrick J. (2024). Accurate structure prediction of biomolecular interactions with AlphaFold 3. Nature.

[38] Varadi M., Anyango S., Deshpande M., Nair S., Natassia C., Yordanova G., Yuan D., Stroe O., Wood G., Laydon A. (2022). AlphaFold Protein Structure Database: Massively expanding the structural coverage of protein-sequence space with high-accuracy models. Nucleic Acids Res..

[39] Chen Z., Yang H., Pavletich N.P. (2008). Mechanism of homologous recombination from the RecA-ssDNA/dsDNA structures. Nature.

[40] Halgren T.A. (2009). Identifying and characterizing binding sites and assessing druggability. J. Chem. Inf. Model..

[41] Friesner R.A., Banks J.L., Murphy R.B., Halgren T.A., Klicic J.J., Mainz D.T., Repasky M.P., Knoll E.H., Shelley M., Perry J.K. (2004). Glide: A new approach for rapid, accurate docking and scoring. 1. Method and assessment of docking accuracy. J. Med. Chem..

[42] Genheden S., Ryde U. (2015). The MM/PBSA and MM/GBSA methods to estimate ligand-binding affinities. Expert. Opin. Drug Discov..

[43] Ediriweera M.K., Tennekoon K.H., Samarakoon S.R. (2019). In vitro assays and techniques utilized in anticancer drug discovery. J. Appl. Toxicol..

[44] Munteanu I.G., Apetrei C. (2021). Analytical Methods Used in Determining Antioxidant Activity: A Review. Int. J. Mol. Sci..

[45] Kumar N., Goel N. (2019). Phenolic acids: Natural versatile molecules with promising therapeutic applications. Biotechnol. Rep..

[46] Kabanda M.M. (2012). Antioxidant activity of rooperol investigated through Cu (I and II) chelation ability and the hydrogen transfer mechanism: A DFT study. Chem. Res. Toxicol..

[47] Parr R.G., Szentpály L.v., Liu S. (1999). Electrophilicity Index. J. Am. Chem. Soc..

[48] Karadag A., Ozcelik B., Saner S. (2009). Review of Methods to Determine Antioxidant Capacities. Food Anal. Methods.

[49] LoPachin R.M., Geohagen B.C., Nordstroem L.U. (2019). Mechanisms of soft and hard electrophile toxicities. Toxicology.

[50] Okuzono S., Ishimura M., Kanno S., Sonoda M., Kaku N., Motomura Y., Nishio H., Oba U., Hanada M., Fukushi J.I. (2018). Streptococcus pyogenes-purpura fulminans as an invasive form of group A streptococcal infection. Ann. Clin. Microbiol. Antimicrob..

[51] Gergova R., Boyanov V., Muhtarova A., Alexandrova A. (2024). A Review of the Impact of Streptococcal Infections and Antimicrobial Resistance on Human Health. Antibiotics.

[52] Ciriaco F., Gambacorta N., Trisciuzzi D., Nicolotti O. (2022). PLATO: A Predictive Drug Discovery Web Platform for Efficient Target Fishing and Bioactivity Profiling of Small Molecules. Int. J. Mol. Sci..

[53] Ciriaco F., Gambacorta N., Alberga D., Nicolotti O. (2021). Quantitative Polypharmacology Profiling Based on a Multifingerprint Similarity Predictive Approach. J. Chem. Inf. Model..

[54] Alberga D., Trisciuzzi D., Montaruli M., Leonetti F., Mangiatordi G.F., Nicolotti O. (2019). A New Approach for Drug Target and Bioactivity Prediction: The Multifingerprint Similarity Search Algorithm (MuSSeL). J. Chem. Inf. Model..

[55] Mishra S., Mazumdar P.A., Dey J., Das A.K. (2003). Molecular modeling of RecX reveals its mode of interaction with RecA. Biochem. Biophys. Res. Commun..

[56] Zhou Z., Pan Q., Lv X., Yuan J., Zhang Y., Zhang M.X., Ke M., Mo X.M., Xie Y.L., Liu Y. (2021). Structural insights into the inhibition of bacterial RecA by naphthalene polysulfonated compounds. iScience.

[57] Kiran K., Patil K.N. (2024). Gallic acid inhibits Staphylococcus aureus RecA protein functions: Role in countering antibiotic resistance in bacteria. J. Appl. Microbiol..

[58] Meanwell N.A. (2011). Improving Drug Candidates by Design: A Focus on Physicochemical Properties As a Means of Improving Compound Disposition and Safety. Chem. Res. Toxicol..

[59] Lipinski C.A., Lombardo F., Dominy B.W., Feeney P.J. (2001). Experimental and computational approaches to estimate solubility and permeability in drug discovery and development settings1PII of original article: S0169-409X(96)00423-1. The article was originally published in Advanced Drug Delivery Reviews 23 (1997) 3–25.1. Adv. Drug Deliv. Rev..

[60] Veber D.F., Johnson S.R., Cheng H.-Y., Smith B.R., Ward K.W., Kopple K.D. (2002). Molecular Properties That Influence the Oral Bioavailability of Drug Candidates. J. Med. Chem..

