# Supplementary material for: Experimental and In Silico Studies to Unravel the Antioxidant and Antibacterial Properties of Lichen Metabolites from Pseudocyphellaria compar and Pseudocyphellaria nudata
Source: Antioxidants (Basel). 2025 Dec 25;15(1):34. doi: 10.3390/antiox15010034 (PMC12837165; doi:10.3390/antiox15010034)
Supplement: Supplementary file 1 [file antioxidants-15-00034-s001.zip › antioxidants-4048202-supplementary.pdf]

Supplementary Material

Experimental and In Silico Studies to Unravel the Antioxidant and Antibacterial Properties of Lichen Metabolites from *Pseudocyphellaria compar* and *Pseudocyphellaria nudata*

Mauricio A. Cuellar [1,2], Jessica Mejía [3], Helena Quintero-Pertuz [4], Alejandro Castro-Álvarez [5,6], Marco Mellado [7], Waleska Vera-Quezada [1,2], Gloria Montenegro [3], Christian Espinosa-Bustos [8], Raquel Bridi [4,*] and Cristian O. Salas [9,*]

1 Facultad de Farmacia, Escuela de Química y Farmacia, Universidad de Valparaíso, Valparaíso 2340000, Chile; mauricio.cuellar@uv.cl (M.A.C.); waleska.vera@uv.cl (W.V.-Q.)
2 Centro de Investigación, Desarrollo e Innovación de Productos Bioactivos (CINBIO), Universidad de Valparaíso, Valparaíso 2340000, Chile
3 Departamento de Ciencias Vegetales, Facultad de Agronomía e Ingeniería Forestal, Pontificia Universidad Católica de Chile, Santiago 7820436, Chile; jcmejia@uc.cl (J.M.); gmonten@uc.cl (G.M.)
4 Departamento de Química Farmacológica y Toxicológica, Facultad de Ciencias Químicas y Farmacéuticas, Universidad de Chile, Santiago 8380000, Chile; helena.quintero@ug.uchile.cl
5 Departamento de Ciencias Preclínicas, Facultad de Medicina, Universidad de La Frontera, Temuco 4780000, Chile; luis.castro@ufrontera.cl
6 Millennium Nucleus Bioproducts, Genomics and Environmental Microbiology (BioGEM), Valparaíso 2390123, Chile
7 Centro de Investigación en Ingeniería de Materiales, Universidad Central de Chile, Santiago 8330507, Chile; marco.mellado@ucentral.cl
8 Departamento de Farmacia, Facultad de Química y de Farmacia, Pontificia Universidad Católica de Chile, Santiago 7820436, Chile; ccespino@uc.cl
9 Medicinal Chemistry Laboratory, Facultad de Química y de Farmacia, Pontificia Universidad Católica de Chile, Santiago 7820436, Chile
* Correspondence: raquelbridi@ciq.uchile.cl (R.B.); cosalas@uc.cl (C.O.S.)

Index

IR, ^{1}H, and ^{13}C NMR spectra of selected compounds…..pags 2 - 11
Table S1. Energy differences between the ground state and radical state of compound **VI** …………………..pag 12
Table S2. Energy differences between the ground state and radical state of compound **VII**……………..…....pag 12
Table S3. Energy differences between the ground state and radical state of compound **VIII**..……..………....pag 12
Table S4. Energy differences between the ground state and radical state of compound **X.**………………..…..pag 13
Table S5. Energy differences between the ground state and radical state of chromophores used in DPPH and ABTS assays..……………………………..………………………………………………………………………...…...pag 13
Figure S1. Structures of the metabolites analyzed and the energy of each molecule in its ground state and radical state studied as an antioxidant agent based on the HAT model, together with the chromophores used for the tests.………………………………………………………………………………………………..………pag 13
Figure S2. Molecular orbitals (HOMO and LUMO), energies and energy differences of the most active (**IX**) and least active (**X**) compounds together Fe–TPTZ complex based in a SET mechanism……………………….……..pag 14
Table S6. Values obtained with SiteMap……………………………………………………………….………...pag 15
Figure S3. Potential binding sites. (**A**) Secondary structure of the RecA monomeric unit, (**B**) the surface of the protein……………………………………………………………………………………………………..………pag 15
Figure S4. RecA protein monomer (blue) and physciosporin (represented by yellow spheres) at the different binding sites obtained with SiteMap…………………………………….……………………………….………pag 16

Figure S5. Graphical representation of the trimer model with a transparent surface to highlight (circled) the regions corresponding to the ATPase domain. ……………………………………………...…………………..….pag 16
Figure S6. Representation of all poses obtained with molecular docking. ………………………………………pag 17

IR spectrum of Pseudocyphellarine A (**VIII**)

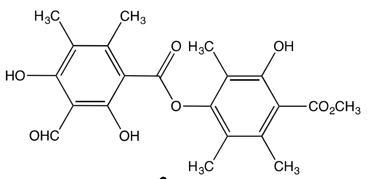

2

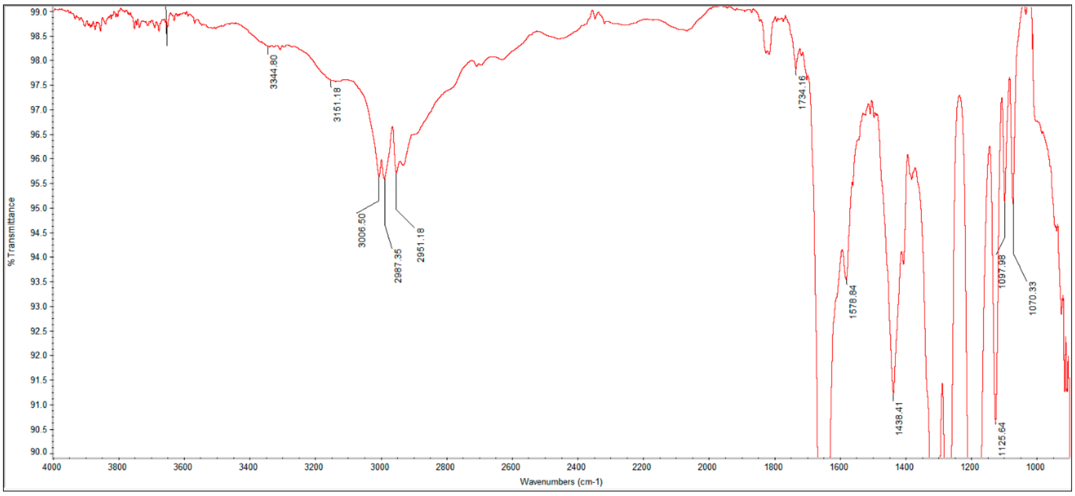

^{1}HNMR spectrum of Pseudocyphellarine A (**VIII**)

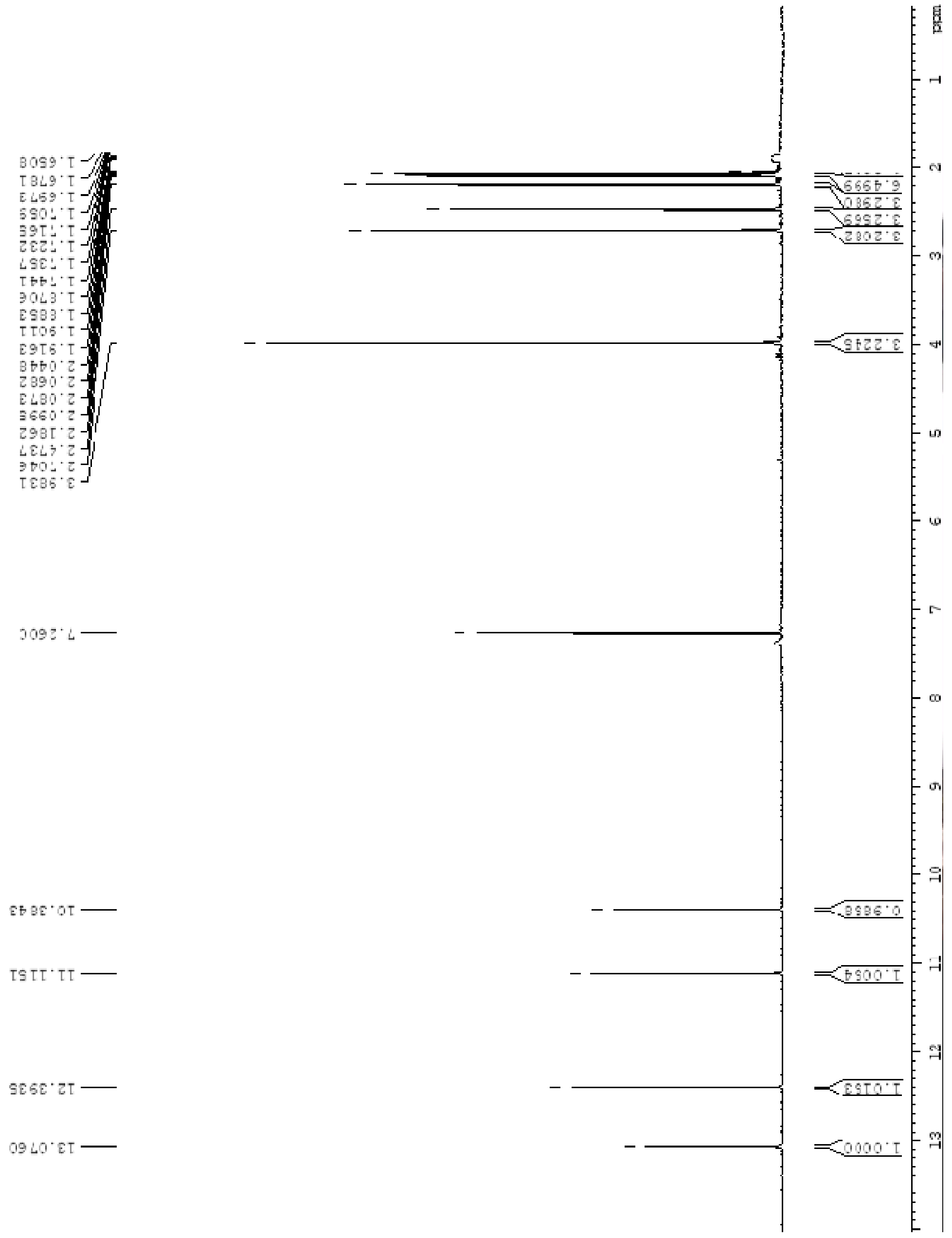

^{13}C NMR spectrum of Pseudocyphellarine A (**VIII**)

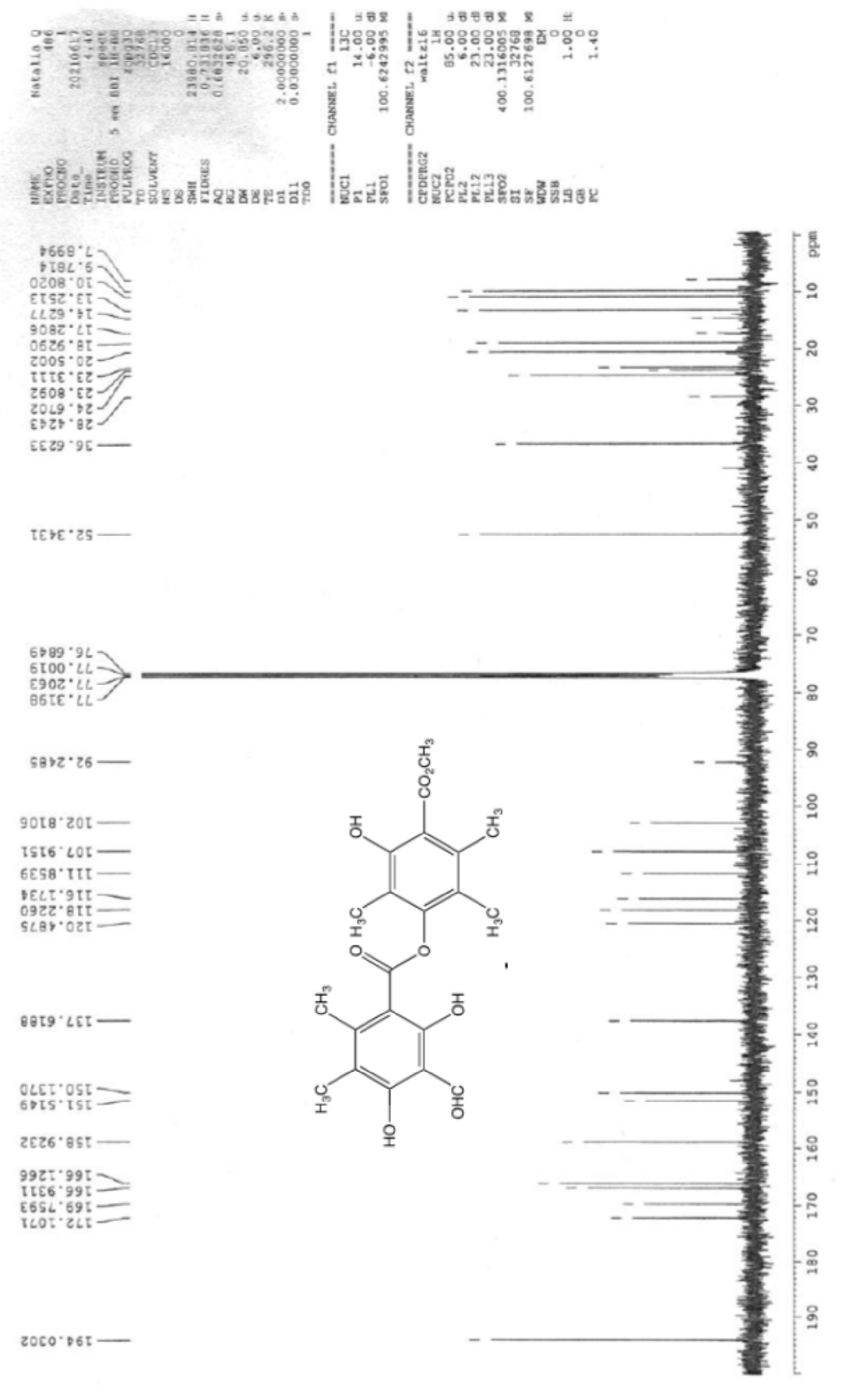

IR spectrum of Physciosporin (**IX**)

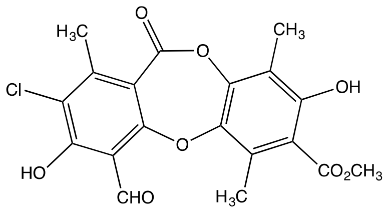

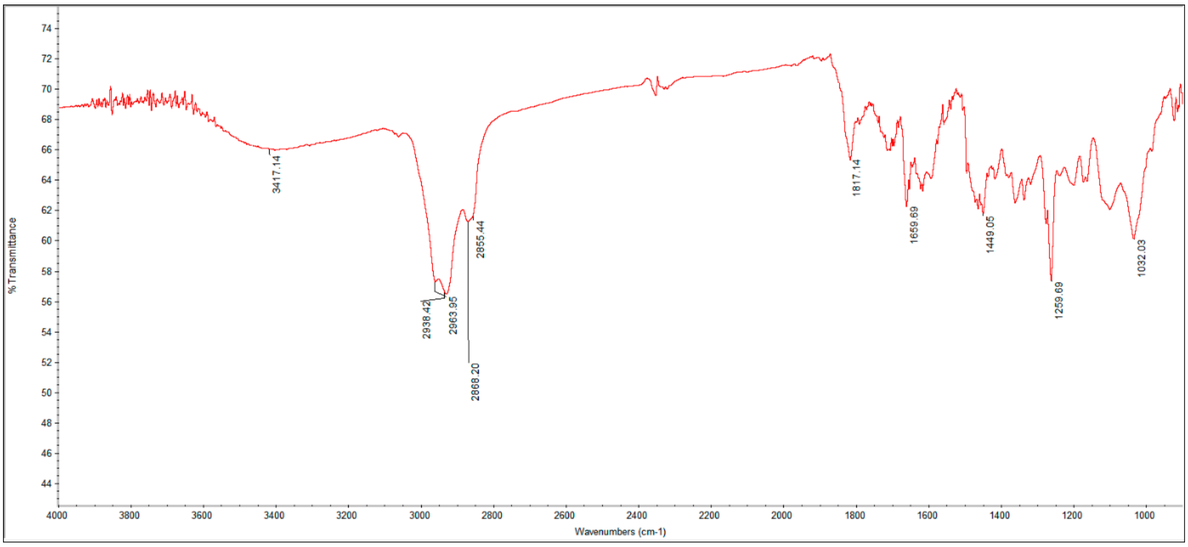

^{1}HNMR spectrum of Physciosporin (**IX**)

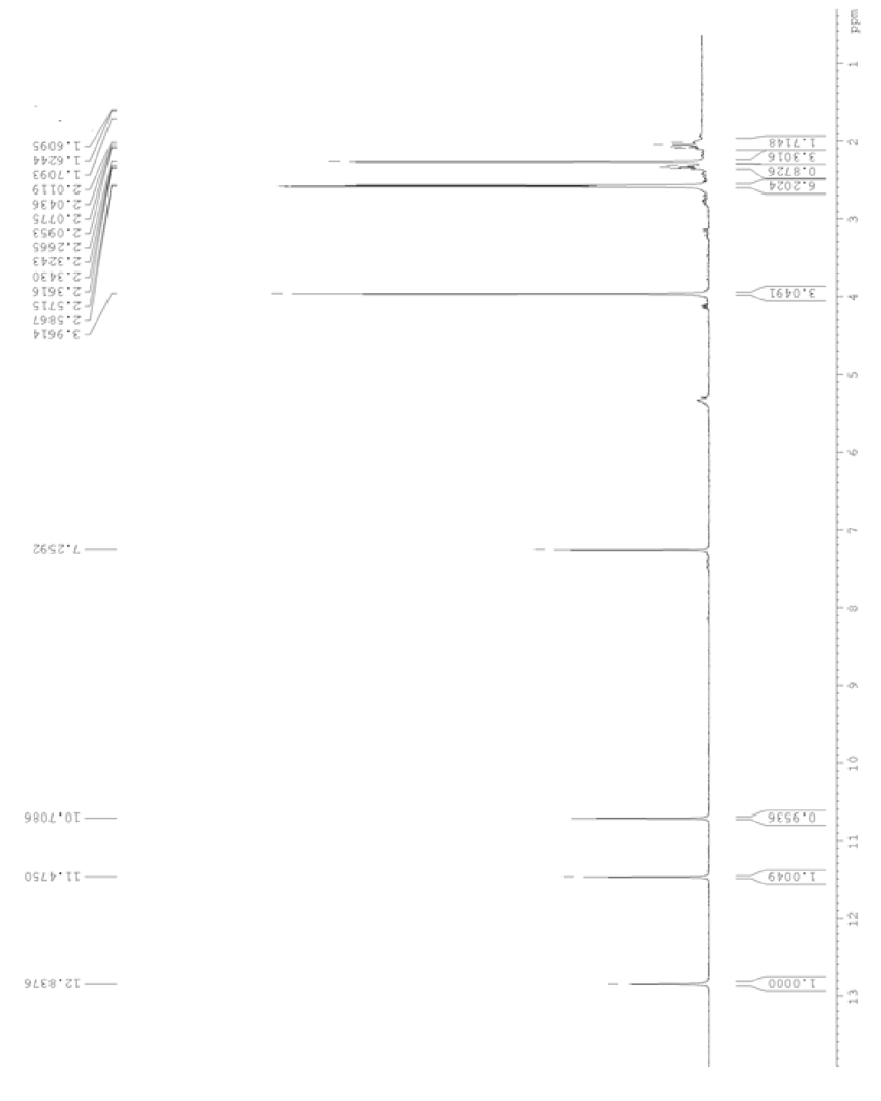

^{13}C NMR spectrum of Physciosporin (**IX**)

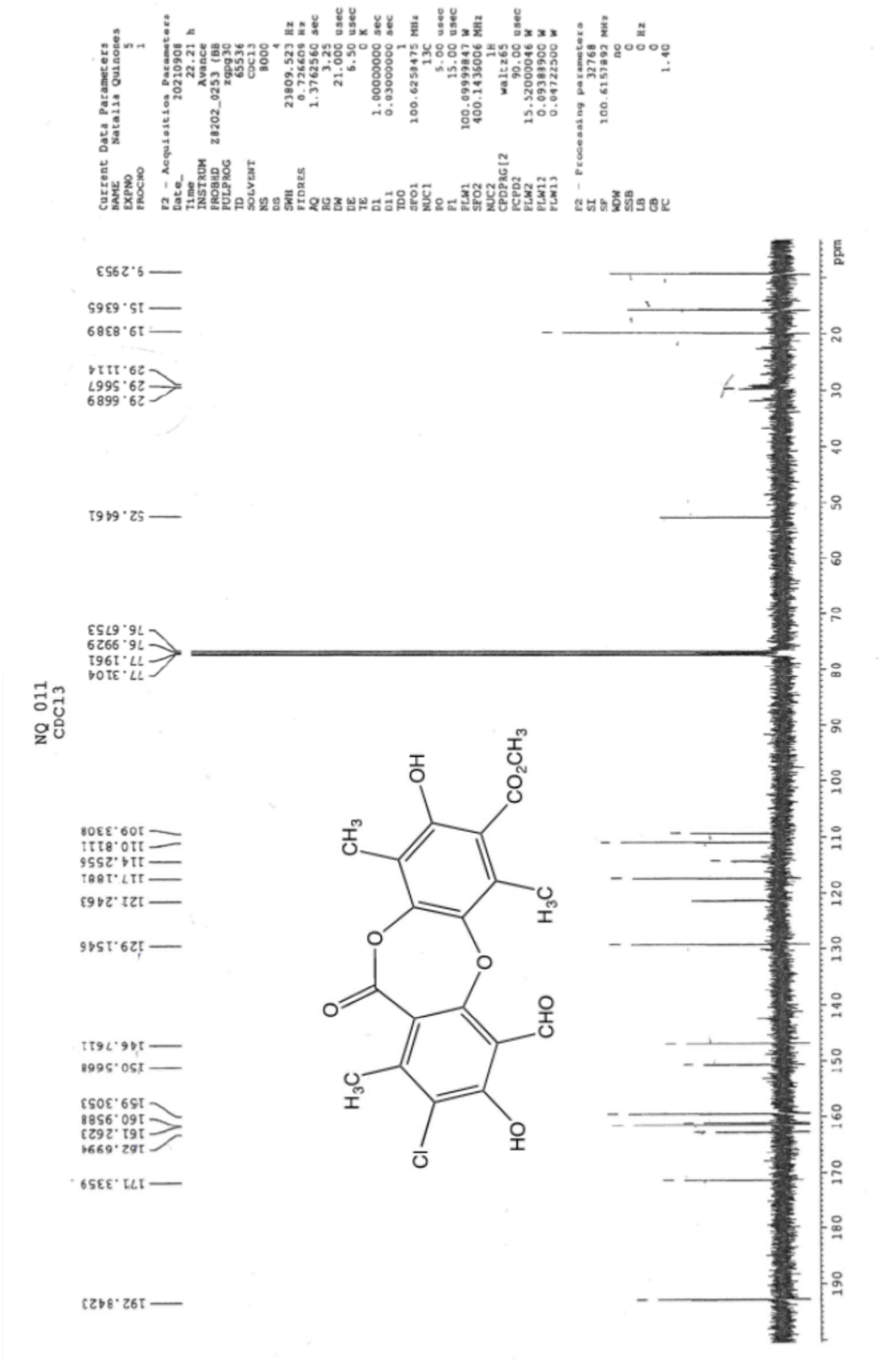

^{1}HNMR spectrum of methyl orsellinate **VII**

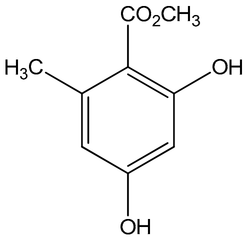

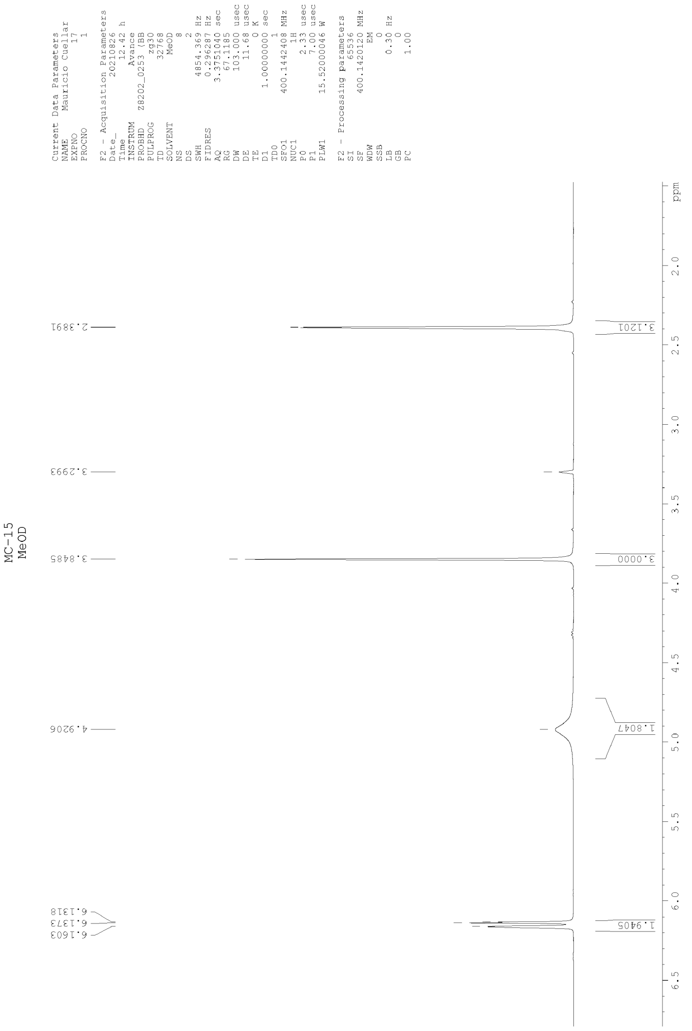

^{13}C NMR spectrum of methyl orsellinate **VII**

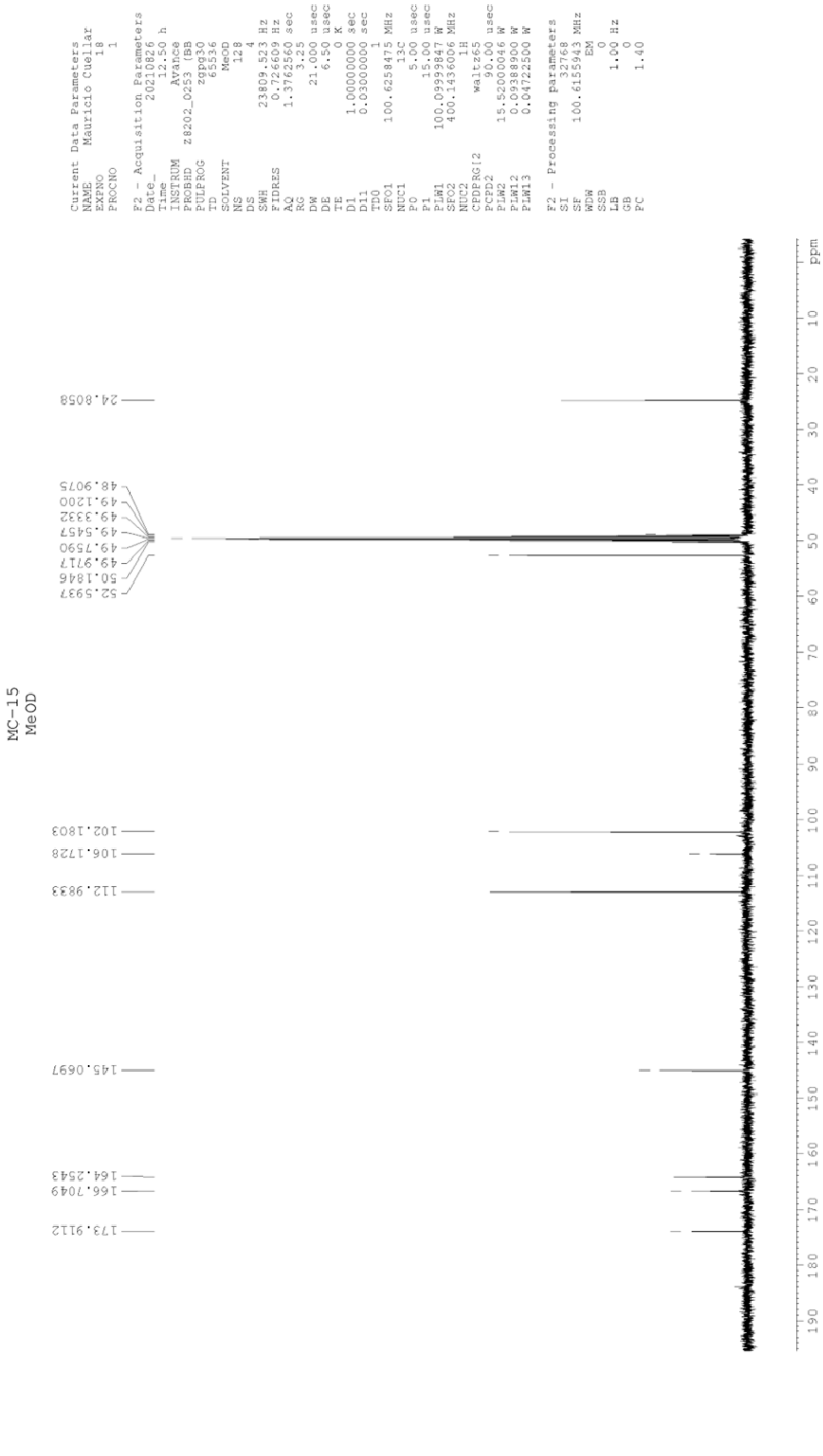

^{1}HNMR spectrum of monomer **X**

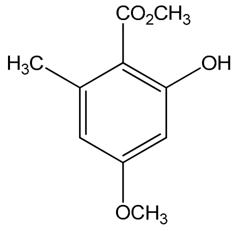

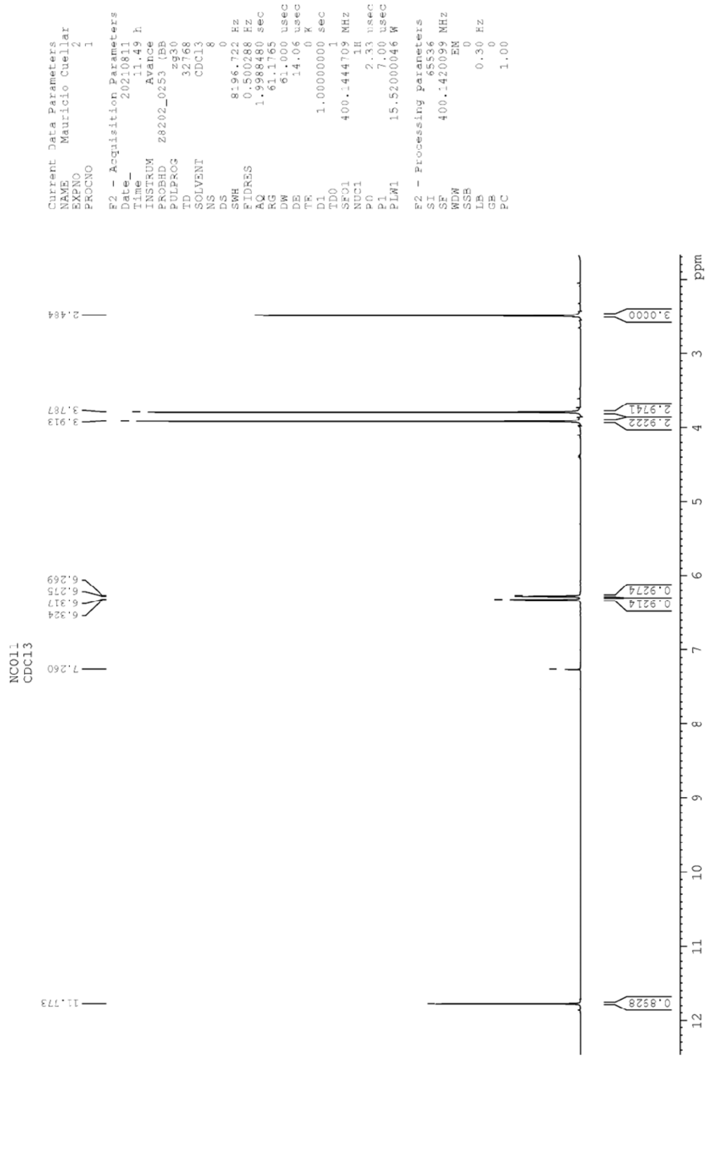

^{13}C NMR spectrum of monomer **X**

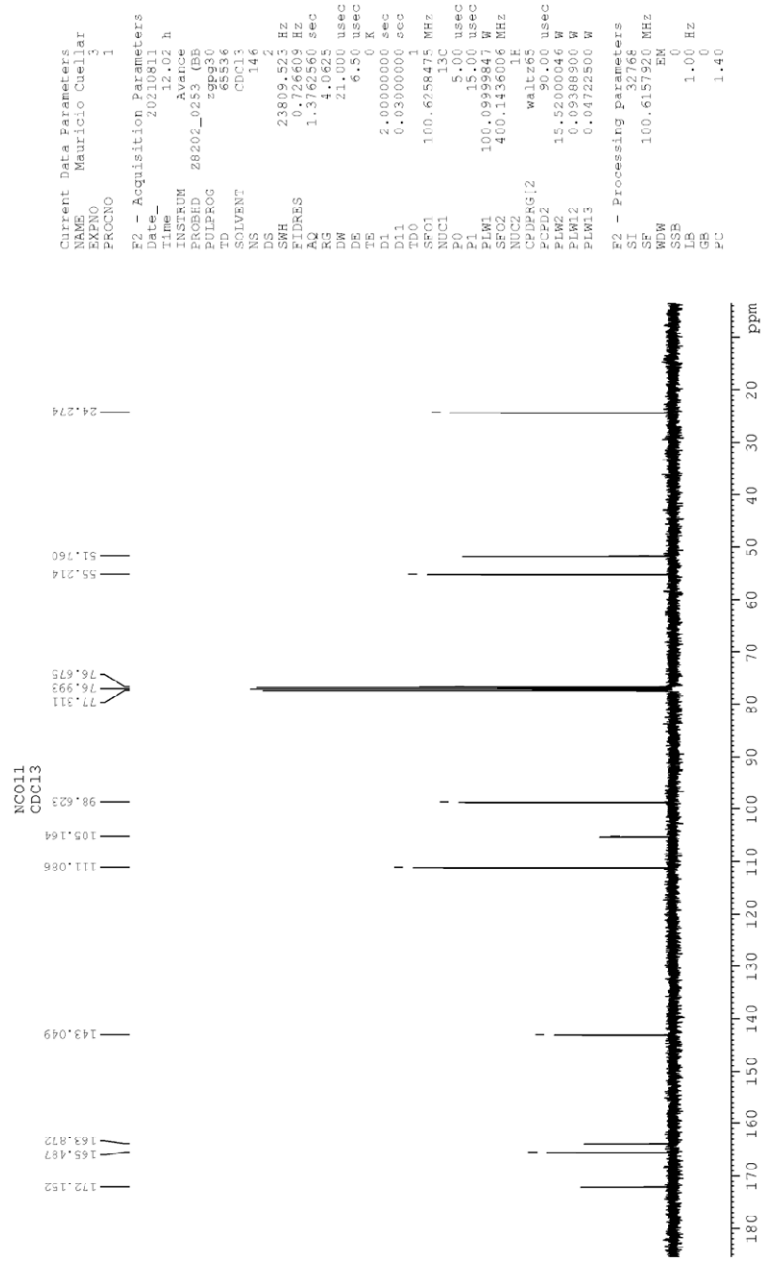

Table S1. Energy differences between the ground state and radical state of compound **VI.**

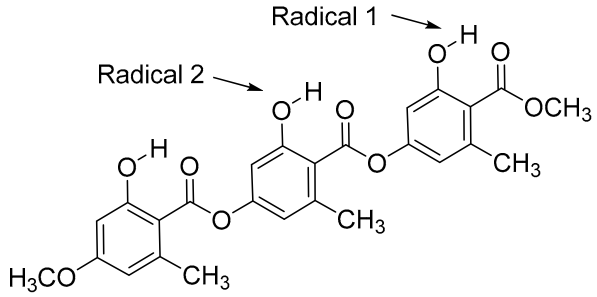

	Ground State Energy	Radical State Energy	Energy Difference
**Radical 1**	-1758.0113	-1757.350678	0.66063694
**Radical 2**		-1757.35203	0.65928658

Table S2. Energy differences between the ground state and radical state of compound **VII.**

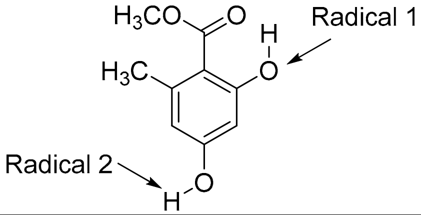

	Ground State Energy	Radical State Energy	Energy Difference
**Radical 1**	-650.08811705	-649.4270903	0.66102679
**Radical 2**		-649.4347338	0.65338328

Table S3. Energy differences between the ground state and radical state of compound **VIII.**

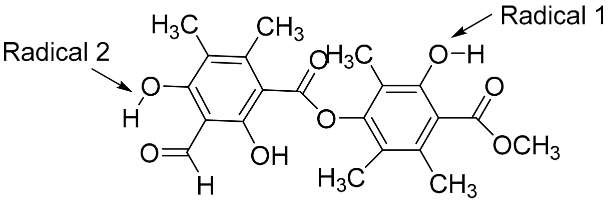

	Ground State Energy	Radical State Energy	Energy Difference
**Radical 1**	-1415.69620365	-1414.172863	1.523341144
**Radical 2**		-1415.050612	0.64559189

Table S4. Energy differences between the ground state and radical state of compound **X.**

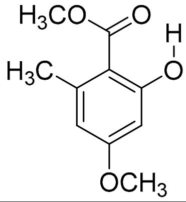

	Ground State Energy	Radical State Energy	Energy Difference
**Radical 1**	-689.2381654	-688.5832767	0.65488866

Table S5. Energy differences between the ground state and radical state of chromophores used in DPPH and ABTS assays.

	Ground State Energy	Radical State Energy	Energy Difference
**ABTS˙**	-2960.188946	-2959.946132	0.24281359
**DPPH˙**	-1418.893809	-1418.260834	0.63297455

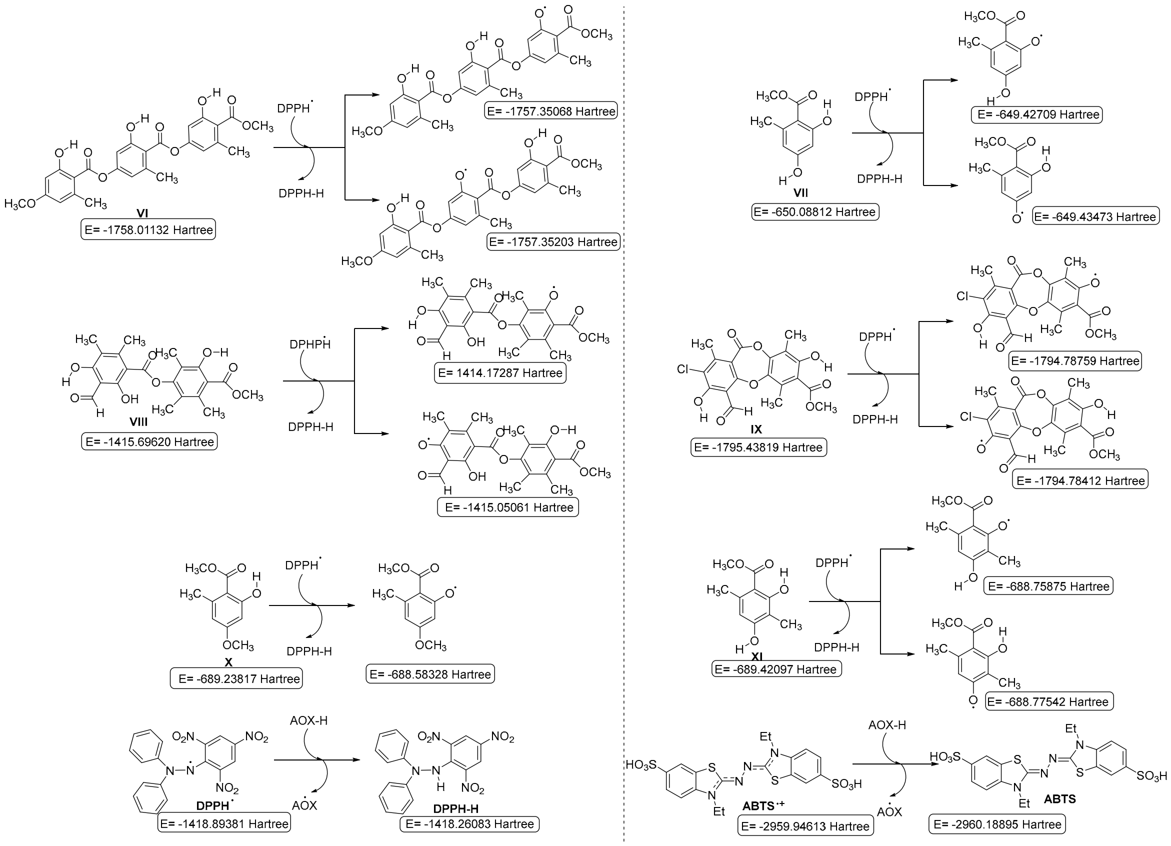

Figure S1. Structures of the metabolites analyzed and the energy of each molecule in its ground state and radical state studied as an antioxidant agent based on the HAT model, together with the chromophores used for the tests.

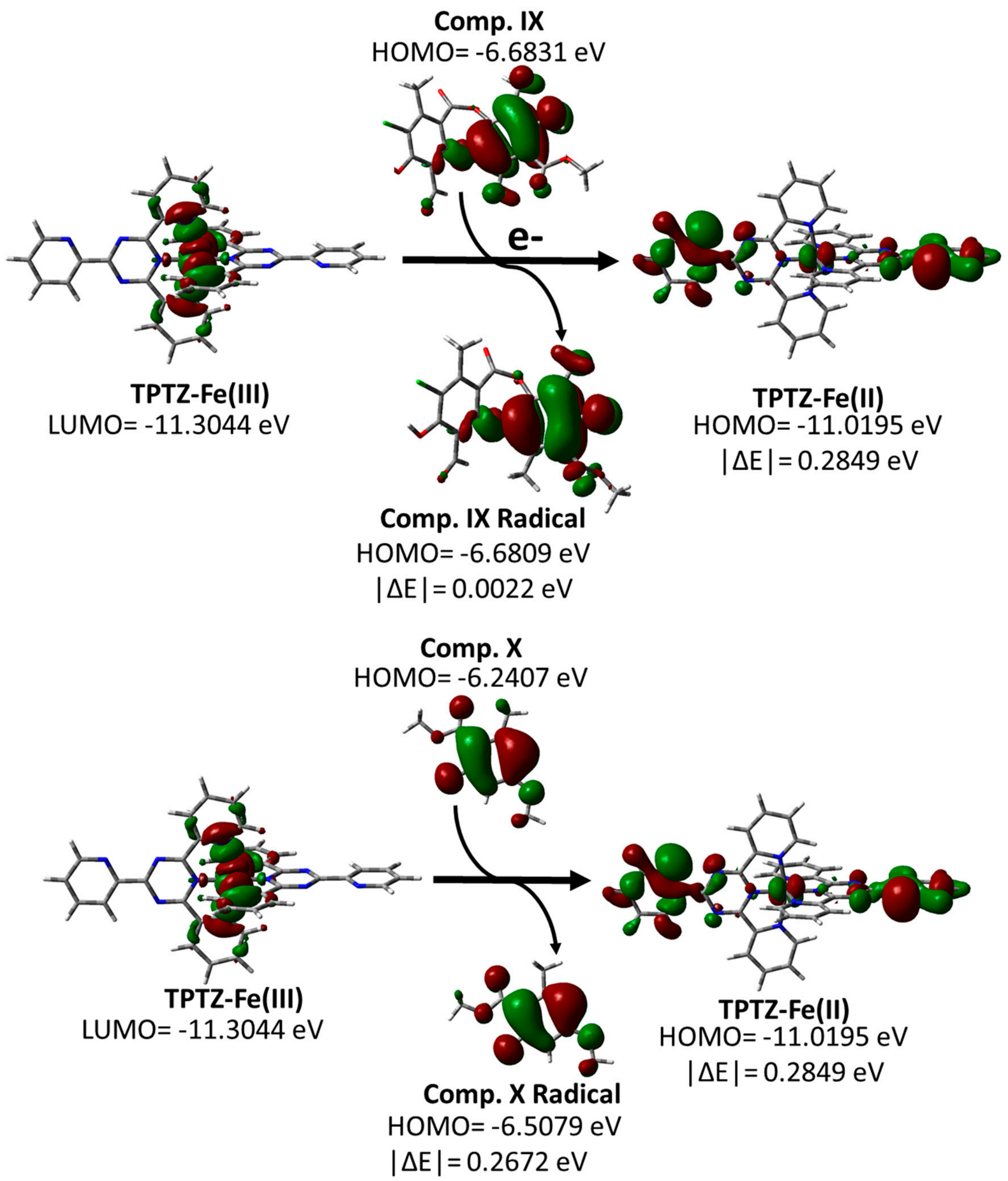

Figure S2. Molecular orbitals (HOMO and LUMO), energies and energy differences of the most active (**IX**) and least active (**X**) compounds together Fe–TPTZ complex based in a SET mechanism.

SiteMap

The SiteMap program determines potential binding sites in a protein, basing its analysis on cavity morphology, size, and volume. The probability of interaction of these sites with a ligand is evaluated using SiteScore, which expresses the probability of binding based on interaction with polar amino acids, considering that values greater than 0.8 identify a promising site; and the Druggability Score (DScore), which estimates the probability of binding of a small ligand by focusing on the hydrophobicity of the pocket. The complementarity of these two values is essential for the rigorous assignment of the most likely binding sites.

Table S6. Values obtained with SiteMap.

Site	SiteScore	Size	DScore	Volume
1	0,854	70	0,787	240,100
2	0,871	57	0,855	112,161
3	0,670	43	0,598	123,480
4	0,719	42	0,703	123,137
5	0,711	35	0,693	128,165

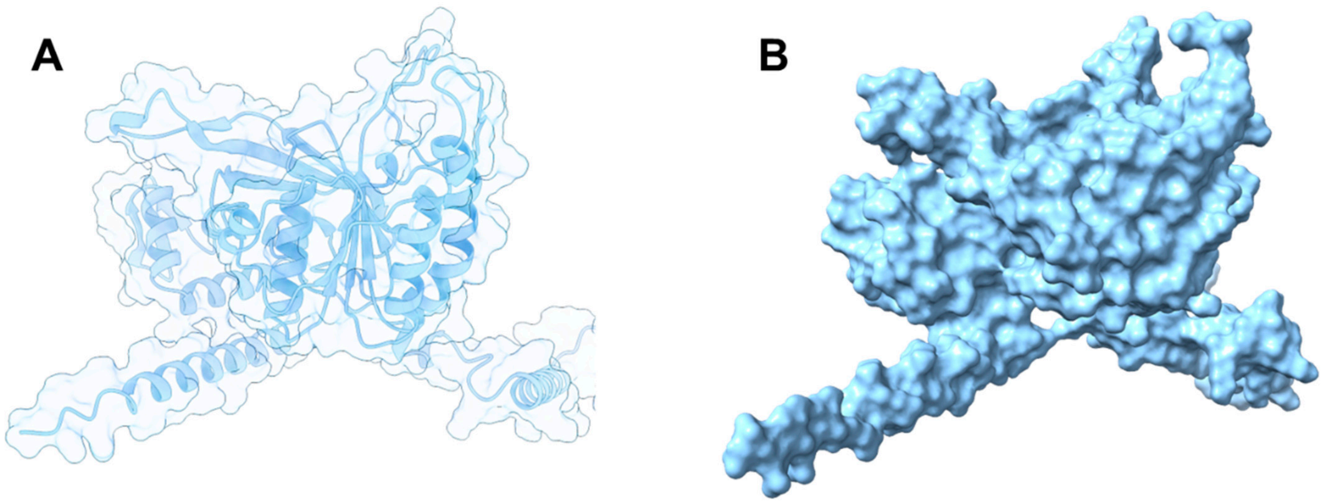

Figure S3. Potential binding sites. (**A**) Secondary structure of the RecA monomeric unit, (**B**) the surface of the protein.

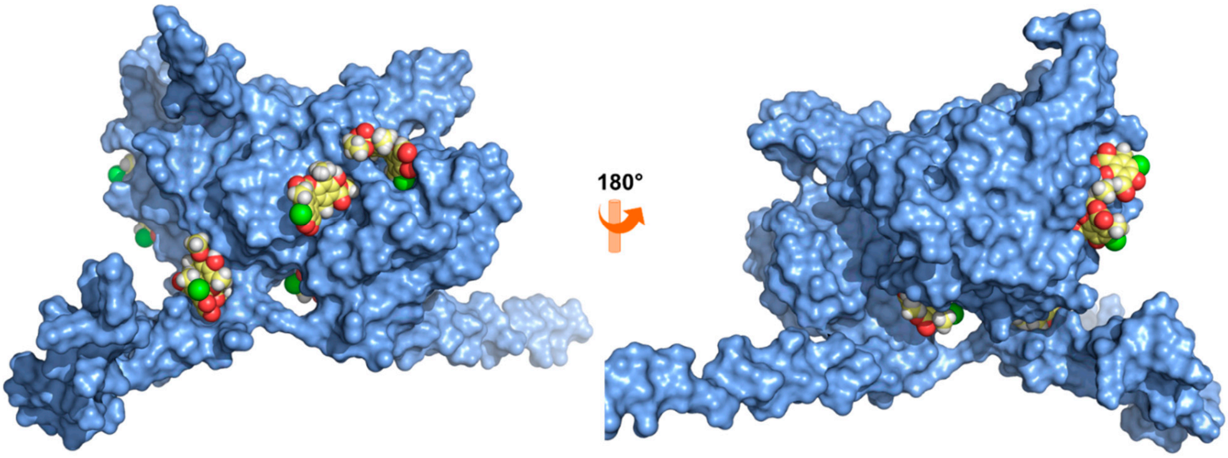

Figure S4. RecA protein monomer (blue) and physciosporin (represented by yellow spheres) at the different binding sites obtained with SiteMap.

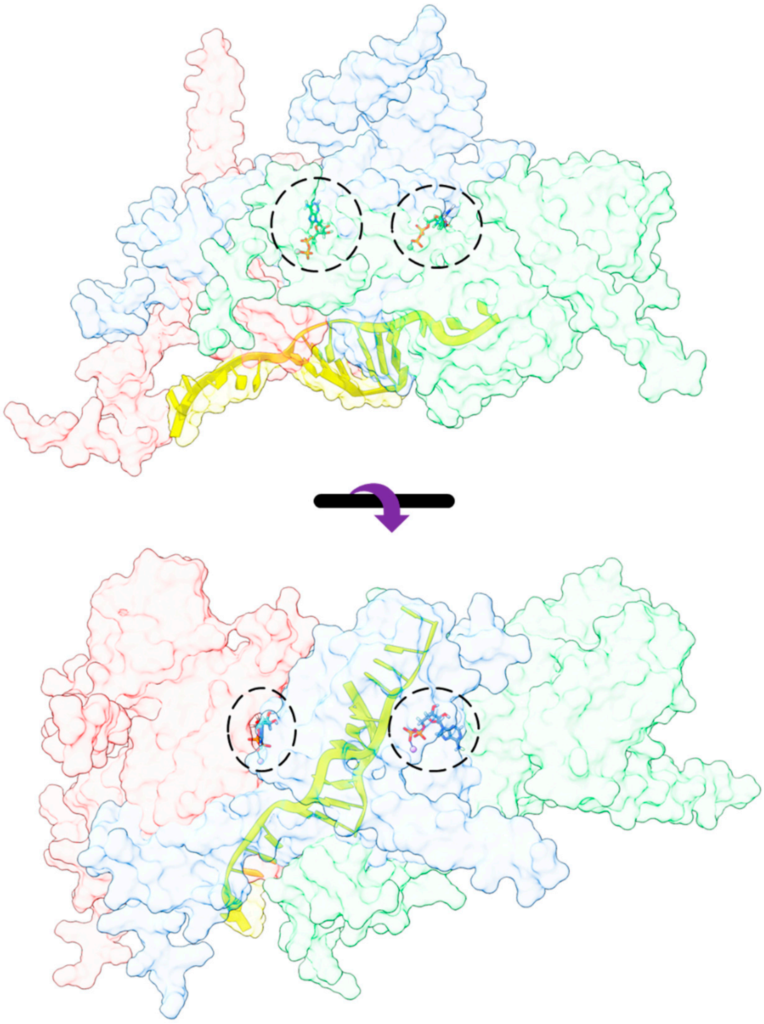

Figure S5. Graphical representation of the trimer model with a transparent surface to highlight (circled) the regions corresponding to the ATPase domain.

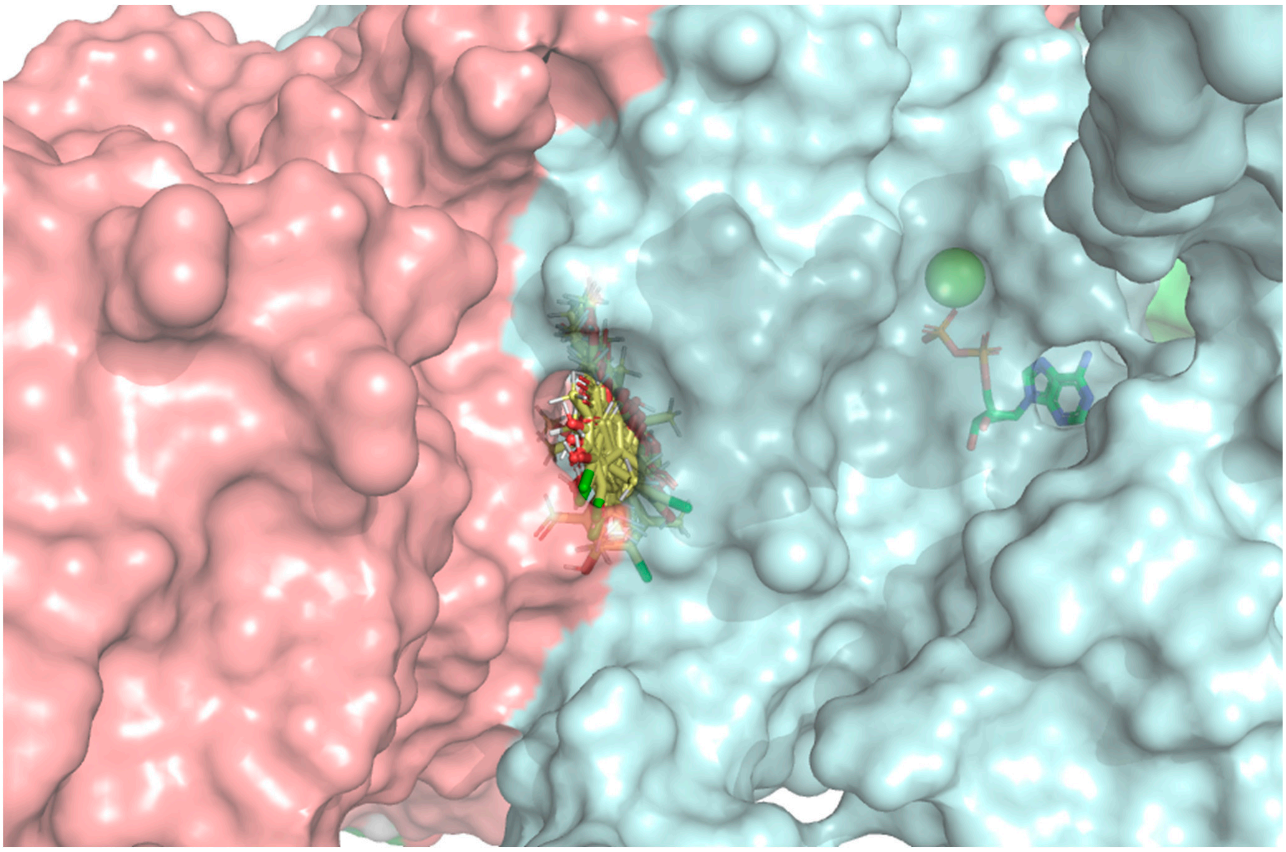

Figure S6. Representation of all poses obtained with molecular docking.
